# Optimising dynamic treatment regimens using sequential multiple assignment randomised trials data with missing data

**DOI:** 10.1186/s12874-025-02595-1

**Published:** 2025-07-01

**Authors:** Jessica Xu, Anurika P. De Silva, Katherine J. Lee, Robert K. Mahar, Julie A. Simpson

**Affiliations:** 1 Centre for Epidemiology and Biostatistics, Melbourne School of Population and Global Health, University of Melbourne, Melbourne, VIC Australia; 2 Clinical Epidemiology and Biostatistics Unit, Murdoch Childrens Research Institute, Royal Children’s Hospital, Melbourne, VIC Australia; 3 Department of Paediatrics, University of Melbourne, Melbourne, VIC Australia; 4 Nuffield Department of Medicine, University of Oxford, Oxford, UK

**Keywords:** Sequential multiple assignment randomised trials, Missing data, Q-learning, Multiple imputation

## Abstract

Dynamic treatment regimens are commonly used for patients with chronic or progressive medical conditions. Sequential multiple assignment randomised trials (SMARTs) are studies used to optimise dynamic treatment regimens by repeatedly randomising participants to treatments. Q-learning, a stage-wise regression-based method used to analyse SMARTs, uses backward induction to compare treatments administered as a sequence. Missing data is a common problem in randomised trials and can be complex in SMARTs given the sequential randomisation. Common methods for handling missing data such as complete case analysis (CCA) and multiple imputation (MI) have been widely explored in single-stage randomised trials, however, the only study that explored these methods in SMARTs did not consider Q-learning. We evaluated the performance of CCA and MI on the estimation of Q-learning parameters in a SMART. We simulated 1000 datasets of 500 participants, based on a SMART with two stages, under different missing data scenarios defined by missing directed acyclic graphs (m-DAGS), percentages of missing data (20%, 40%), stage 2 treatment effects, and strengths of association with missingness in stage 2 treatment, patient history and outcome. We also compared CCA and MI using retrospective data from a longitudinal smoking cessation SMART. When there was no treatment effect at either stage 1 or 2, we observed close to zero absolute bias in the stage 1 treatment effect and similar empirical standard errors for CCA and MI under all missing data scenarios. When all participants had a relatively large stage 2 treatment effect, we observed minimal bias from both CCA and MI, with slightly greater bias for MI. Empirical standard errors were higher for MI compared to CCA under all scenarios except for when data were missing not dependent on any variables. When the stage 2 treatment effect varied between participants and data were missing dependent on other variables (for example, stage 1 responder status missing dependent on stage 1 treatment and baseline variables), we observed greater bias for MI when estimating the stage 1 treatment effect, which increased with the percentage missingness, while the bias for CCA remained minimal. Resulting empirical standard errors were lower or similar for MI compared to CCA under all missing data scenarios. Results showed that for a two-stage SMART, MI failed to capture the differences between treatment effects when the stage 2 treatment effect varied between participants.

**Supplementary Information:**

The online version contains supplementary material available at 10.1186/s12874-025-02595-1.

## Background

Clinicians must often make multi-stage dynamic decisions when treating patients with chronic or progressive diseases such as cancer [1] or mental health disorders [2]. Dynamic treatment regimens (DTRs) are a set of sequential treatment rules commonly used to guide these treatment decisions [3]. Sequential multiple assignment randomised trials (SMARTs) are clinical studies with multiple stages of randomisation [4] that can be used to estimate the value of DTRs and thus optimise sequential treatment decisions.

Missing data are inevitable in many randomised controlled trials (RCTs) and primarily occur in the outcome variable(s). In SMARTs, missing data can be more complicated due to the multiple stages of randomisation where data collection in subsequent stages depends on treatment allocation and participant outcomes in previous stages. For example, in a two-stage SMART, if a participant drops out during stage 1, then no data would be collected at stage 2 including treatment allocation and the outcome.

One common method for analysing SMART data is Q-learning. Q-learning is a stage-wise method that uses regression models to estimate the quality of a treatment decision at each treatment stage while appropriately accounting for previous decisions, participant outcome(s), and other baseline variables [5]. An advantage of using Q-learning to analyse SMART data is that it accounts for delayed effects from previous stages by starting the analysis from the final stage and working backwards.

Selecting an appropriate statistical method for handling the missing data that is based on pre-specified missing data assumptions is important [6]. Common methods to handle missing data include complete case analysis (CCA) and multiple imputation (MI). Only participants who have complete data for all analysis variables are included in a CCA [7]. MI is a two-stage process that repeatedly imputes missing values based on the observed data, then performs the target analysis on each imputed dataset, and combines the resulting parameter estimates and standard errors while accounting for the uncertainties associated with the missing values [8]. Although the performance of CCA and MI have been widely explored in conventional, single stage randomised trials [9], only a single study, led by Shortreed et al. [10], has explored how well these methods perform in a SMART setting when weighted regression (weights used to adjust for unequal randomisation probabilities) was used to estimate the mean response for each treatment regime. Shortreed et al. identified multiple challenges associated with using imputation methods for handling missing data in SMARTs [10], and pointed to the need for further research to understand how MI methods perform when the underlying missing data assumptions are violated.

Because Q-learning is a complex analytic approach using backward stepwise regression of the multiple stages of SMART designs, it complicates how missing data should be handled. For example, when using MI, compatibility between the target analysis and imputation models is known to be important [11], but the backward induction nature of Q-learning means that correctly defining the imputation method is not straight forward.

In this paper, we aim to evaluate the performance of CCA and MI on the estimation of Q-learning parameters in a typical two-stage, two-treatment SMART. We begin by formalising several missing data mechanisms that might be found in a SMART context and reviewing Q-learning, before describing in detail the simulation study following the guidelines in Morris et al. [12]. We then present the results of the simulation study which compares the performance of CCA and MI for estimation of the stage 1 treatment effect under a range of missingness proportions, missing data mechanisms and varied stage 2 treatment effects. We also compare the different analytical approaches in a case study, using data from a smoking cessation SMART.

## Methods

### Missingness in SMART designs

**Fig. 1:**
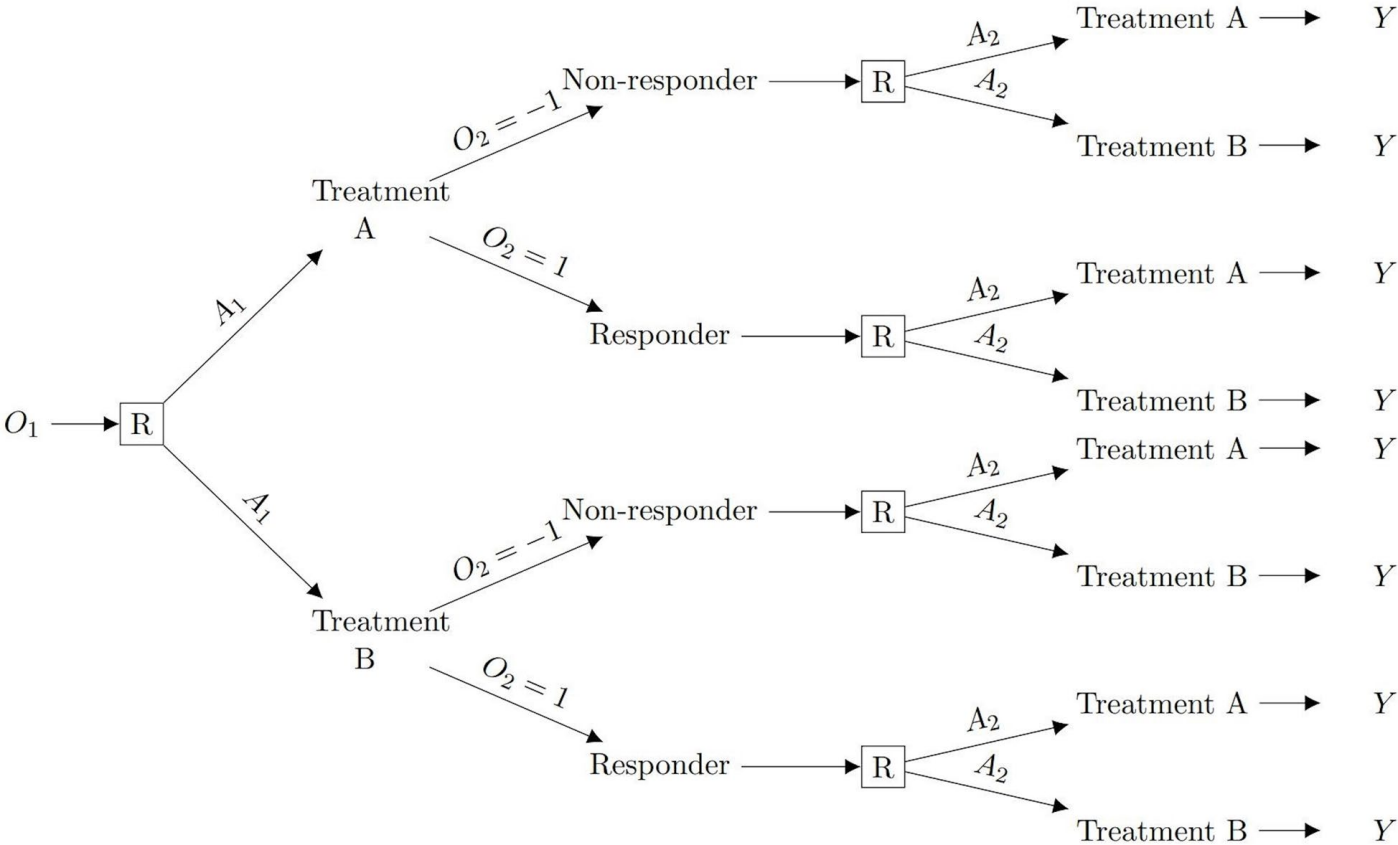
Example of a SMART design where participants are first randomised in stage 1 and re-randomised in stage 2. Footnotes: ^a^ ${O}_{1}$ represents a baseline characteristic; ${A}_{1}$ and ${A}_{2}$ represent treatment at stage 1 and stage 2 respectively; $O_2$ represents stage 1 responder status; and $Y$ represents the outcome after stage 2 treatment (also the final outcome). R is randomisation at each stage. ^b^ For simplicity, we present the tree diagram for those with ${O}_{1}=1$, this is repeated for those with ${O}_{1}=-1$

A simple two-stage SMART design where randomisation at stage 2 is not dependent on the response to stage 1 is presented in Fig. 1. In this design we denote a single (binary) baseline characteristic by ${O}_{1}=\{-\text{1,1}\}$; treatments at stage 1 and stage 2 by ${A}_{1}=\{-\text{1,1}\}$ and ${A}_{2}=\{-\text{1,1}\}$ respectively; stage 1 responder status by ${O}_{2}=\{-\text{1,1}\}$; and the continuous outcome after stage 2 treatment by $Y$.

**Fig. 2:**
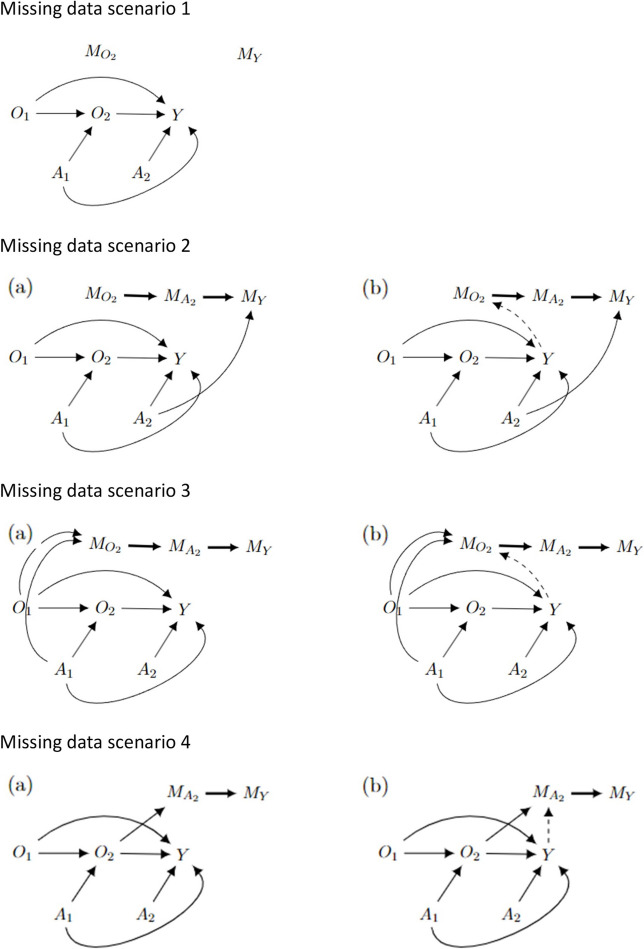
m-DAGs depicting a two-stage SMART where the nodes ${M}_{z}$ represent missingness in variable $z$. Footnotes: ^a^ Nodes ${M}_{O2}$, ${M}_{A2}$ and ${M}_{Y}$ represent missingness in $O_2$, ${A}_{2}$ and $Y$ respectively. ^b^ When ${O}_{2}$ is missing, both ${A}_{2}$ and $Y$ are also missing as denoted by a thick black arrow. Graphs (b) are complements to (a) that include a dashed arrow representing missingness due to a common cause between $Y$ and ${M}_{O2}$ or ${M}_{A2}$ which induces an association. ^C^ For missing data scenario 1) ${O}_{2}$ and $Y$ are missing not dependent on any variables; 2a) ${O}_{2}$ is missing not dependent on other variables and $Y$ missing dependent on ${A}_{2}$; 2b) ${O}_{2}$ missing dependent on $Y$ and $Y$ missing dependent on ${A}_{2}$; 3a) ${O}_{2}$ missing dependent on ${A}_{1}$ and $O_1$; 3b) $O_2$ missing dependent on ${A}_{1}$, ${O}_{1}$ and $Y$; 4a) ${A}_{2}$ missing dependent on $O_2$; 4b) ${A}_{2}$ missing dependent on $O_2$ and $Y$

We begin by formalising four missingness scenarios that we might observe within a typical two-stage, two-treatment SMART design depicted using missing data directed acyclic graphs (m-DAGs, Fig. 2). The nodes ${M}_{O2}$, ${M}_{A2}$ and ${M}_{Y}$ in the m-DAG [6, 13] represent missingness in ${O}_{2}$, ${A}_{2}$ and $Y$ respectively. We assume that ${O}_{1}$ and ${A}_{1}$ are complete.

- Missing data scenario 1: Data records could be lost post data collection at both stages, which would lead to stage 1 responder status (${O}_{2}$) and stage 2 outcome ($Y$) to be missing not dependent on any variables, but treatment at stage 2 (${A}_{2}$) being fully observed.
- Missing data scenario 2: The final outcome ($Y$) could be missing dependent on the treatment given in stage 2 (${A}_{2}$). For example, participants allocated to a less effective treatment arm could be more likely to drop out before the outcome data are collected. In addition to having missing data in the outcome, we could also have missingness in the stage 1 responder status (${O}_{2}$) where (a) ${O}_{2}$ is missing not dependent on any variables; and (b) ${O}_{2}$ is missing due to a common cause between ${M}_{O2}$ and $Y$ which induces an association.
- Missing data scenario 3: Participants could drop out after stage 1 leading to missing responder status in stage 1 (${O}_{2}$), missing treatment assignment in stage 2 (${A}_{2}$), and missing outcome data in stage 2 ($Y$). This could occur for many reasons including not being satisfied with the treatments, potential side effects or baseline characteristics (i.e. older participants being less likely to continue). Reasons for drop out could be related to (a) stage 1 treatment (${A}_{1}$) and baseline characteristics (${O}_{1}$) or (b) due to ${A}_{1}$, ${O}_{1}$ and also a common cause between ${M}_{O2}$ and $Y$ which induces an association.
- Missing data scenario 4: Participants who were responders to stage 1 treatment may be more likely to not agree to be randomised at stage 2 and therefore have missing treatment assignment (${A}_{2}$) and outcome ($Y$) at stage 2. In this scenario, ${A}_{2}$ could be missing dependent on (a) ${O}_{2}$ or (b) ${O}_{2}$ and also a common cause between ${M}_{A2}$ and $Y$ which induces an association.

We chose to use m-DAGs to depict the missingness assumptions instead of the standard Rubin classification (MCAR/MAR/MNAR) because as stated by Lee et al. [6] the standard Rubin classifications are difficult to assess when there are missing data in multiple variables. For trials that consist of multiple stages such as SMARTs, there are often missing data in multiple variables (as depicted in our missing data scenarios described above) where a participant does not complete stage 1 and progress to stage 2 of the trial.

#### Q-learning

Q-learning is a stage-wise method where regression models are used to estimate the quality of each treatment decision at each stage using Q-functions, while accounting for variables from previous stages [5].

For a two-staged SMART, the stages are defined by $k,$ where $k=1, 2$. Here, ${O}_{k}$ defines the variables observed before the $k$ th stage; ${A}_{k}$ is the treatment assigned at the $k$ th stage where ${A}_{k} = \{-1, 1\}$; ${H}_{k}$ is the history at the $k$ th stage where ${H}_{1} = {O}_{1}$ and ${H}_{2} = ({O}_{1}, {A}_{1}, {O}_{2})$; and ${Y}_{k}$ is the outcome at the end of the $k$ th stage.

A DTR is a sequence of treatment decision rules. A two-stage DTR consists of two decisions $({d}_{1}, {d}_{2})$ which depend on the history at that stage:

1 $${d}_{k}({h}_{k}) \in {A}_{k}, k = 1, 2$$

The Q-functions, ${Q}_{k}$, represent the expected value of the outcome conditional on treatment and history for the two stages and are defined as follows [14]:

2 $${Q}_{2}({H}_{2}, {A}_{2}) = E[{Y}_{2}|{H}_{2}, {A}_{2}]$$

3 $${Q}_{1}\left({H}_{1}, {A}_{1}\right)= E\left[{Y}_{1} + \underset{{a}_{2}}{\text{max}}{Q}_{2}({H}_{2}, {a}_{2})|{H}_{1}, {A}_{1}\right]$$

If the Q-functions defined above were known, then the optimal DTR for stage 1 and 2 would be:

4 $${d}_{k}^{opt} ({h}_{k}) = {\text{arg}} \underset{{a}_{k}}{\text{max}}{Q}_{k}\left({h}_{k}, {a}_{k}\right), k = 1, 2$$

However, in reality the true values of the Q-functions are unknown and therefore we use linear regression models to estimate the Q-functions:

5 $${Q}_{k}\left({H}_{k}, {A}_{k}; {\beta }_{k}, {\psi }_{k}\right)= {\beta }_{k}^{T} {H}_{k} + \left({\psi }_{k}^{T} {H}_{k}\right){A}_{k}, k = 1, 2,$$

where ${\beta }_{k}$ and ${\psi }_{k}$ are the regression coefficients that represent the effects of the history variables and the interaction between history variables and treatment, respectively.

For Q-learning, the optimal decision rule for the second stage is first identified and then subsequently incorporated into the first stage expectation, resulting in each Q-function being estimated via backward induction [2]. For example, for a two-stage design with a continuous primary outcome at each stage, linear regression is performed to determine the optimal decision rule at stage 2 first, based on the primary continuous outcome ${Y}_{2}$ and history $({H}_{2}, {A}_{2})$ to obtain $( {\widehat{\beta }}_{2}, {\widehat{\psi }}_{2})$:

6 $${\widehat{d}}_{2}^{opt} ({h}_{2}) = {\text{arg}} \underset{{a}_{2}}{\text{max}}{Q}_{2}\left({h}_{2}, {A}_{2}; {\widehat{\beta }}_{2}, {\widehat{\psi }}_{2}\right) = {\text{sign}}({\widehat{\psi }}_{2}^{T}{h}_{2})$$

Then we obtain an estimated outcome for stage 1 assuming that the optimal second stage outcome would be realised. This stage-1 outcome that combines data and expectations (often referred to as a pseudo-outcome) is defined as:

7 $${\widehat{Y}}_{1i} = {Y}_{1i} + \underset{{a}_{2}}{\text{max}}{Q}_{2}({H}_{2}, {a}_{2}; {\widehat{\beta }}_{2}, {\widehat{\psi }}_{2})= {Y}_{1i} + {\widehat{\beta }}_{2}^{T} {H}_{2i} + \left| {\widehat{\psi }}_{2}^{T}{H}_{2i}\right|, i = 1, ..., n$$

for the $i$ th patient ($i=1$ to $n$). Note that we can specify ${Y}_{1i} = 0$ if we only analyse one single primary outcome at the end of stage 2 (end of the study for both stages).

Regression is then performed at stage 1, based on the stage-1 pseudo-outcome ${\widehat{Y}}_{1i}$ and history $({H}_{1}, {A}_{1})$ to obtain $\left({\widehat{\beta }}_{1}, {\widehat{\psi }}_{1}\right)$, leading to the estimated first stage optimal decision rule, as defined below:

8 $${\widehat{d}}_{1}^{opt} ({h}_{1}) = {\text{arg}} \underset{{a}_{1}}{\text{max}}{Q}_{1}\left({h}_{1}, {A}_{1}; {\widehat{\beta }}_{1}, {\widehat{\psi }}_{1}\right) = {\text{sign}}({\widehat{\psi }}_{1}^{T}{h}_{1})$$

In a SMART the interest is typically in which treatment will give the best outcome at the end of the study, represented by the $\psi$ parameters. For a two-staged trial the estimated optimal DTR is $({\widehat{d}}_{1}^{opt}, {\widehat{d}}_{2}^{opt})$.

When we use Q-learning to analyse SMART data, an estimation issue, commonly referred to as ‘non-regularity', can occur when at least some participants have no unique optimal treatment decision after stage 1, which can induce bias in the treatment effect estimation (see Additional file 2). In our simulation study described in the following section, we considered scenarios where this issue does and does not arise.

### Simulation study

We used a simulation study to evaluate the performance of CCA and MI for handling missing data in a simple SMART to determine the optimal DTR using Q-learning.

Chakraborty et al. [14] proposed six SMART simulation settings which are either ‘regular’, ‘near-regular’ or ‘non-regular’ (see Additional file 2 for a description of these simulation settings). We considered the same simulation settings as it is known that non-regularity affects the estimation of certain Q-learning parameters (treatment effect parameters at any stage prior to the last). These simulation settings have since been used in several methodological studies [15–18]. We use three of Chakraborty’s simulation settings in our simulation study.

#### Data generation

We generated 1000 simulated datasets of $n=500$ each using the data-generating models described below:

1. Participants were randomly assigned a treatment (binary) at stage 1 with the probability of 0.5.
  9 $$P ({A}_{1} = 1) = P ({A}_{1} = -1) = 0.5$$
2. A binary baseline characteristic ${O}_{1}$, was generated with the probability of 0.5.
  10 $$P ({O}_{1} = 1) = P ({O}_{1} = -1) = 0.5$$
3. A binary stage 1 responder status ${O}_{2}$ was generated with the following probability, conditional on ${O}_{1}$ and ${A}_{1}$:
  11 $$P [{O}_{2} = 1|{O}_{1}, {A}_{1}] = 1 - P [{O}_{2} = -1|{O}_{1}, {A}_{1}] = expit({\delta }_{1}{O}_{1} + {\delta }_{2}{A}_{1})$$
  where $${\text{expit}}(x) = {\text{exp}}(x)/(1 + {\text{exp}}(x))$$.
  A participant is considered a responder if ${O}_{2} = 1$ and a non-responder if ${O}_{2} = -1$ 
4. Participants’ treatment allocation at stage 2 was generated as a binary variable with a probability of 0.5, with stage 1 treatment and responder status as stratification variables, that is, within each ${A}_{1}$ and ${O}_{2}$ stratum:
  12 $$P ({A}_{2} = 1) = P ({A}_{2} = -1) = 0.5$$
5. Finally, a stage 2 continuous outcome was generated as ${\mu }_{Y2} =E(Y|{O}_{1}, {A}_{1}, {O}_{2}, {A}_{2})$ 
  13 $${\mu }_{Y} = {\gamma }_{1} + {\gamma }_{2}{O}_{1} + {\gamma }_{3}{A}_{1} + {\gamma }_{4}{O}_{1}{A}_{1} + {\gamma }_{5}{A}_{2} + {\gamma }_{6}{O}_{2}{A}_{2} + {\gamma }_{7}{A}_{1}{A}_{2} + \varepsilon$$
  where ε ∼ N (0, 1).

We considered 5 simulation settings that investigated a range of stage 1 and stage 2 treatment effects and represented different non-regularity settings, including simulation settings 1, 3 and 4 proposed by Chakraborty et al. [14] (corresponds to Treatment effect 1, 3 and 4 respectively) and two extra settings (Treatment effect 2 and 5) where stage 1 treatment effect did not equal 0:

- Treatment effect 1: (fully non-regular) there is no treatment effect on $Y$ at either stage 1 or 2 for any participant.
- Treatment effect 2: (fully regular) there is a relatively large treatment effect on $Y$ at stage 2 for every participant, and a treatment effect at stage 1 with a magnitude of –0.5 (see Table 1).
- Treatment effect 3: (non-regular) there is no treatment effect on $Y$ at stage 2 for half of the participants, but a relatively large effect for the other half, and no treatment effect at stage 1.
- Treatment effect 4: (regular, but close to non-regular) there is a very weak treatment effect on $Y$ at stage 2 for half of the participants, but a relatively large effect for the other half, and minimal treatment effect at stage 1.
- Treatment effect 5: (non-regular) there is no treatment effect on $Y$ at stage 2 for half of the participants, but a relatively large effect for the other half, and a treatment effect at stage 1 with a magnitude of 0.5 (see Table 1). This simulation setting is similar to Treatment effect 3 but we assume that there is an even larger treatment effect at stage 2 for the other half.

**Table 1:** Parameter values used to generate the 5 different non-regularity settings

Treatment effect	Stage 1 responder status (${{\varvec{O}}}_{2}$)^a^		Outcome (${\varvec{Y}}$)^b^							Stage 1 treatment effect
	${\delta }_{1}$	${\delta }_{2}$	${\gamma }_{1}$	${\gamma }_{2}$	${\gamma }_{3}$	${\gamma }_{4}$	${\gamma }_{5}$	${\gamma }_{6}$	${\gamma }_{7}$	${\psi }_{10}$
**1**	0.5	0.5	0	0	0	0	0	0	0	0
**2**	0.5	0.5	0	0	−0.5	0	0	0	1	−0.5
**3**	0.5	0.5	0	0	−0.5	0	0.5	0	0.5	0
**4**	0.5	0.5	0	0	−0.5	0	0.5	0	0.49	−0.01
**5**	0.5	0.5	0	0	−0.5	0	1	0	1	0.5

Footnotes: ^a^ Delta values used to define the probability of ${O}_{2}$

^b^Outcome generated by Eq. 13: ${\mu }_{Y} = {\gamma }_{1} + {\gamma }_{2}{O}_{1} + {\gamma }_{3}{A}_{1} + {\gamma }_{4}{O}_{1}{A}_{1} + {\gamma }_{5}{A}_{2} + {\gamma }_{6}{O}_{2}{A}_{2} + {\gamma }_{7}{A}_{1}{A}_{2} + \varepsilon$ where ε ∼ N (0, 1)

^c^Degree of non-regularity is calculated by the probability of the linear combination ${\gamma }_{5} + {\gamma }_{6}{O}_{2} + {\gamma }_{7}{A}_{1}=0$ where the linear combinations tell us the stage 2 treatment effects for the different combinations of ${O}_{2}$ and ${A}_{1}$

^d^ ${\psi }_{10}$ derived by combinations of the parameters of the generative models, including $\delta$ s used to generate the stage 1 responder status (${O}_{2}$) and $\gamma$ s used to generate the outcome

We dropped simulation setting 2 (regular, but close to non-regular), setting 5 (non-regular) and setting 6 (fully regular) proposed by Chakraborty et al. [14] as they had a similar regularity setting to the settings we considered (Treatment effect 2, 3, 4 and 5). We added two extra simulation settings (Treatment effect 2 and 5) where there was a larger treatment effect at stage 1.

For the 5 simulation settings considered above, the different stage 2 treatment effects depended on their stage 1 responder status and stage 1 treatment assigned, and were achieved by modifying the parameter values used to generate the outcome data $Y$ (Eq. 13). The parameter values are provided in Table 1.

#### Missingness

We set up 4 scenarios with different patterns and percentages of missing data as described below (Fig. 2). The parameter values used in these settings are described in Additional file 1: Table S1. All variables not mentioned in a scenario were considered fully observed.

1. Missing data scenario 1: stage 1 responder status (${O}_{2}$) and stage 2 outcome ($Y$) were set to missing not dependent on any variables.
2. Missing data scenario 2: stage 1 responder status (${O}_{2}$) was set to missing not dependent on any variables. Stage 2 outcome data ($Y$) and treatment at stage 2 (${A}_{2}$) were set to missing whenever there were missing data in ${O}_{2}$. Additionally, stage 2 outcome data ($Y$) were set to missing dependent on ${A}_{2}$ as specified in the following logistic regression model:
  14 $${\text{logit}}[P ({M}_{Y} = 1)] = {\alpha }_{0} + {\alpha }_{1}[{A}_{2} = 1]$$
  We also explored missing data scenario 2b, where we assumed that the missingness in ${O}_{2}$ was dependent on a common cause between ${M}_{O2}$ and $Y$ which induces an association between ${M}_{O2}$ and $Y$, by generating the missingness in ${O}_{2}$ using the following logistic regression model:
  15 $${\text{logit}}[P ({M}_{O2} = 1)] = {\alpha }_{0} + {\alpha }_{1}Y$$
3. Missing data scenario 3: stage 1 responder status (${O}_{2}$) was set to missing using a logistic regression where the probability of missingness was dependent on baseline characteristics (${O}_{1}$) and treatment at stage 1 (${A}_{1}$):
  16 $${\text{logit}}[P ({M}_{02} = 1)] = {\alpha }_{0} + {\alpha }_{1}[{O}_{1} = 1] + {\alpha }_{2}[{A}_{1} = 1]$$
  In this scenario, stage 2 treatment (${A}_{2}$) and the outcome ($Y$) were set to missing whenever there is missing data in stage 1 responder status (${O}_{2}$).
  We also considered missing data scenario 3b where the missingness in ${O}_{2}$ was additionally dependent on a common cause between ${M}_{O2}$ and $Y$ which induces an association between ${M}_{O2}$ and $Y$. Therefore, the missingness in stage 1 responder status (${O}_{2}$) was specified using the following logistic regression model:
  17 $${\text{logit}}[P ({M}_{02} = 1)] = {\alpha }_{0} + {\alpha }_{1}[{O}_{1} = 1] + {\alpha }_{2}[{A}_{1} = 1] + {\alpha }_{3}Y$$
4. Missing data scenario 4: if a participant was a responder to stage 1 treatment (${O}_{2} = 1$) then they were set to be more likely to have missing in stage 2 treatment (${A}_{2}$) and outcome ($Y$). This was achieved using the following logistic regression model:
  18 $${\text{logi}}{\text{t}}[P ({M}_{A2} = 1)] = {\alpha }_{0} + {\alpha }_{1}[{O}_{2} = 1]$$
  We also explored missing data scenario 4b where stage 2 treatment (${A}_{2}$) was set to missing according to the following logistic regression model including the variables ${O}_{2}$ (stage 1 responder status) and $Y$ (assuming a common cause of missingness in ${A}_{2}$ and $Y$):
  19 $${\text{logit}}[P ({M}_{A2} = 1)] = {\alpha }_{0} + {\alpha }_{1}[{O}_{2} = 1] + {\alpha }_{2}Y$$

The $\alpha$ parameters (except ${\alpha }_{0}$) in the equation above were assigned based on the strength of association between the missing indicator and predictors of missingness, namely $\alpha =\text{log}\left(Odds Ratio\right)$. For a weak association an odds ratio of 1.6 was used and for a strong association an odds ratio of 3 was used. The intercepts of the logistic regression models (${\alpha }_{0})$ above were chosen by iteration to achieve the required percentage of missingness (20% or 40%).

For each of the 5 simulation settings mentioned above which represented different stage 2 treatment effects, we considered the 7 missing data scenarios and for all missing data scenarios, except missing data scenario 1, we explored both weak and strong associations between the missing indicator and predictors of missingness. For all missing data scenarios, we also explored both 20% and 40% missingness. In total, we set up 130 simulation scenarios.

#### Estimand of interest and target analysis

Our interest lies in estimating the treatment effect at stage 1 estimated via the parameters of a Q-learning model at stage 1 from the two-stage, two-treatment SMART design.

These parameters were estimated using two-stage Q-learning process. First, we analysed outcome data at stage 2, using the following linear model to find the optimal treatment decision that maximises the ${Q}_{2}$ function:

20 $${Q}_{2}\left({H}_{2}, {A}_{2}\right) = {\beta }_{20} + {\beta }_{21}{O}_{1} + {\beta }_{22}{A}_{1} + {\beta }_{23}{O}_{1}{A}_{1} + \left({\psi }_{20} + {\psi }_{21}{O}_{2} + {\psi }_{22}{A}_{1}\right){A}_{2}$$

Next, we obtained an estimated outcome for stage 1 (known as the stage 1 pseudo-outcome) for each individual, assuming that it is the average best outcome that can be obtained (Eq. 7).

Finally, we fitted a linear model to stage 1 data, based on the stage 1 pseudo-outcome, using the following model:

21 $${Q}_{1}({H}_{1}, {A}_{1}) = {\beta }_{10} + {\beta }_{11}{O}_{1} + \left({\psi }_{10} + {\psi }_{11}{O}_{1}\right){A}_{1}$$

The treatment effect at stage 1 (${\psi }_{10}$), used to estimate the optimal decision rule in stage 1, is the estimand of interest.

We also explored the stage 2 treatment effect estimated via the parameters of the Q-learning model at stage 2 (${\psi }_{20}$).

#### Methods to handle missing data

We compared the performance of MI and CCA for handling the missing data in each of the scenarios. For CCA, all participants with missing values in any of the variables were excluded from the analysis. For MI, the imputation model for $Y$ (stage 2 outcome) included all the variables in the target analysis model, (including the interaction terms ${O}_{1}{A}_{1}$, ${O}_{2}{A}_{2}$ and ${A}_{1}{A}_{2}$). To ensure compatibility with our analysis model, the imputation model for ${O}_{2}$ also included the interaction term $Y{A}_{2}$, and the imputation model for ${A}_{2}$ included the interaction terms $Y{O}_{2}$ and $Y{A}_{1}$. Since the variables that need to be imputed consist of both binary and continuous variables, we used MI by chained equations where different univariate models can be used to impute the different types of variables with missing data [19]. We did not include any auxiliary variables in the imputation model and we return to this in the discussion.

According to the rule of thumb that the number of imputations should be at least equal to the proportion of missing data [20], for the scenarios where approximately 20% of the cases had missing data 20 imputations were generated, and for the scenarios where approximately 40% of the cases had missing data 40 imputations were generated.

#### Performance measures

Performance measures selected to compare the performance of CCA and MI were the absolute bias, the difference between the average estimate over 1000 datasets with the true value of ${\psi }_{10}$; the empirical standard error, derived by square rooting the empirical variance; the mean squared error (MSE), derived by adding the variance and squared bias obtained above; bootstrap model-based standard errors, calculated by obtaining the standard error of the 200 bootstrap samples for each simulated dataset and then averaging these standard errors over the 1000 simulated datasets; and coverage, the proportion of 95% confidence intervals that contain the true value of ${\psi }_{10}$. Due to long computational time, we selected treatment effect simulation settings 1,2 and 4 and for these selected two missing data scenarios (m-DAG 1 and m-DAG 2a) to present the bootstrap model-based standard errors and coverage. All data simulation and analyses were conducted using R version 4.3.1.

### Real data example

To further compare the performance of the CCA and MI, we applied the two missing data methods to a real data example.

The real data example is based on two smoking cessation studies, namely the Project Quit (PQ) study [6] and the Forever Free (FF) study, which combined form a SMART. Both studies were conducted by the Center for Health Communications Research at the University of Michigan. The PQ study aimed to help participants quit smoking by using a web-based smoking cessation program and the FF study was a follow-on study to the PQ study where the purpose was to help participants of the PQ study who quit smoking to remain non-smokers and those who did not quit, quit smoking. Combining data from the two studies, we get a simple two-stage SMART where the PQ study represents the first stage, and the FF study represents the second stage of the SMART.

We analysed this dataset as a SMART using the baseline characteristics (${O}_{1}$) gender, age, health maintenance organisation, education, number of cigarettes smoked, motivation and self-efficacy. Stage 1 intervention was whether the participant was randomised to a web-based smoking cessation program that did or did not include tailored hypothetical success stories. The binary variable collected at the end of stage 1 capturing whether a participant smoked in the last 7 days, was used as the stage 1 responder status (${O}_{2}$). At stage 2 all participants (including both those who had quit smoking and those who had not quit smoking) were randomised to an intervention that requested them to read a specific version of the smoking cessation and relapse prevention booklets, wordings of these booklets were adjusted according to whether the participant had quit smoking or not. In the control group no booklets were handed out. The final outcome ($Y$) collected at the end of stage 2 was the number of attempts to quit smoking.

There were 1848 participants who were randomised to an intervention at stage 1 (PQ intervention). Only 479 participants who gave consent for the FF study were randomised to a treatment at stage 2. To have a dataset with a complete SMART design, our analysis was restricted to the 479 participants that received interventions at both stages. Therefore, by design, there were no participants who dropped out of the study prior to being randomised at stage 2.

## Results

### Simulation Study

Figures 3, 4, 5 and 6 and Additional file 3: Figures S1–S6 summarise the performance of CCA and MI for estimating ${\psi }_{10}$, across the different simulation settings with varied stage 1 and 2 treatment effects and missing data scenarios described above.

**Fig. 3:**
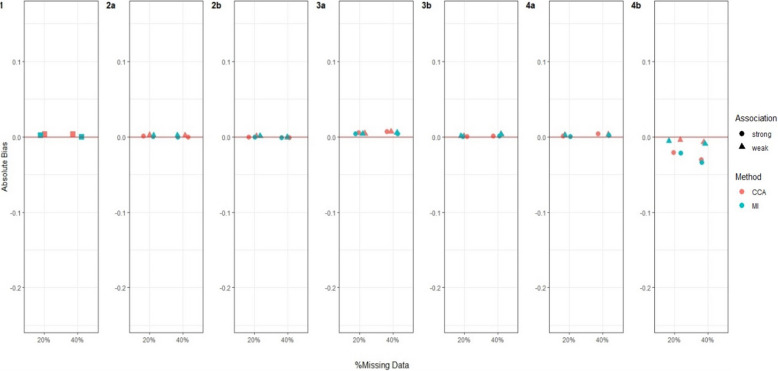
Bias in estimating stage 1 treatment effect ${\psi }_{10}$ under Treatment effect 1 simulation setting^a^. Footnotes: ^a^ True value of ${\psi }_{10}$ = 0 for Treatment effect 1 simulation setting when there is no treatment effect at either stage 1 or 2 for any participant. ^b^ Complete case analysis (CCA) and multiple imputation (MI) were used to handle missing data, where 20% or 40% had incomplete data under the seven missing data scenarios described in the Missingness in SMART designs section, see Fig. 2. ^c^ For weak association between the missing indicator and other variables (as described below) an OR of 1.6 was used; and for a strong association an OR of 3 was used. The other variables were: missing data scenario 2a) ${A}_{2}\to {M}_{Y}$; 2b)$Y\to {O}_{2}$ and ${A}_{2}\to {M}_{Y}$; 3a) ${A}_{1}\to {M}_{O2}$ and ${O}_{1}\to {M}_{O2}$; 3b) ${A}_{1}\to {M}_{O2}$,${O}_{1}\to {M}_{O2}$ and $Y\to {M}_{O2}$; 4a)${O}_{2}\to {M}_{A2}$; and 4b) ${O}_{2}\to {M}_{A2}$ and $Y\to {M}_{A2}$. ^d^ Monte Carlo error less than 0.0035 for all estimates

**Fig. 4:**
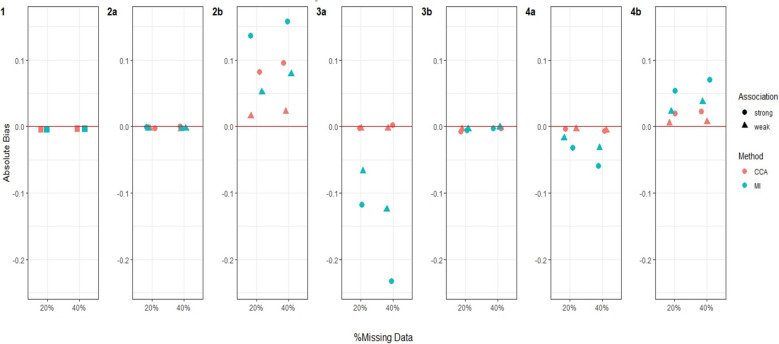
Bias in estimating stage 1 treatment effect ${\psi }_{10}$ under Treatment effect 2 simulation setting^a^. Footnotes: ^a^ True value of ${\psi }_{10}$ = −0.5 for Treatment effect 2 simulation setting when there is a relatively large treatment effect at stage 2 for every participant. ^b^ Complete case analysis (CCA) and multiple imputation (MI) were used to handle missing data, where 20% or 40% had incomplete data under the seven missing data scenarios described in the Missingness in SMART designs section, see Fig. 2. ^c^ For weak association between the missing indicator and other variables (as described below) an OR of 1.6 was used; and for a strong association an OR of 3 was used. The other variables were: missing data scenario 2a) ${A}_{2}\to {M}_{Y}$; 2b)$Y\to {M}_{O2}$ and ${A}_{2}\to {M}_{Y}$; 3a) ${A}_{1}\to {M}_{O2}$ and ${O}_{1}\to {M}_{O2}$; 3b) ${A}_{1}\to {M}_{O2}$,${O}_{1}\to {M}_{O2}$ and $Y\to {M}_{O2}$; 4a)${O}_{2}\to {M}_{A2}$; and 4b) ${O}_{2}\to {M}_{A2}$ and $Y\to {M}_{A2}$. ^d^ Monte Carlo error less than 0.0035 for all estimates

**Fig. 5:**
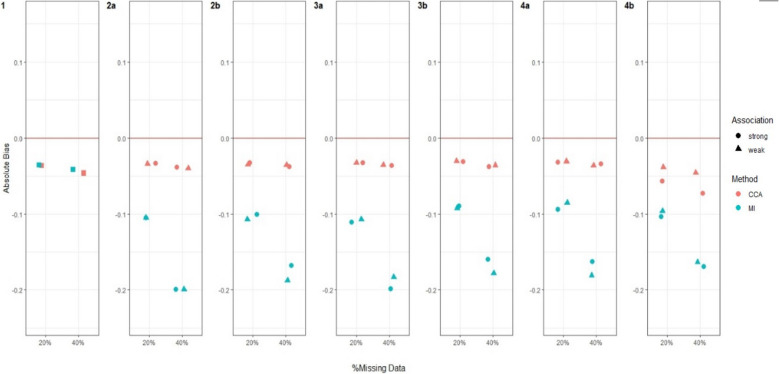
Bias in estimating stage 1 treatment effect ${\psi }_{10}$ under Treatment effect 4 simulation setting^a^. Footnotes: ^a^ True value of ${\psi }_{10}$ = −0.01 for Treatment effect 4 simulation setting when there is a very weak treatment effect at stage 2 for half of the participants, but a relatively large effect for the other half. ^b^ Complete case analysis (CCA) and multiple imputation (MI) were used to handle missing data, where 20% or 40% had incomplete data under the seven missing data scenarios described in the Missingness in SMART designs section, see Fig. 2. ^c^ For weak association between the missing indicator and other variables (as described below) an OR of 1.6 was used; and for a strong association an OR of 3 was used. The other variables were: missing data scenario 2a) ${A}_{2}\to {M}_{Y}$; 2b)$Y\to {M}_{O2}$ and ${A}_{2}\to {M}_{Y}$; 3a) ${A}_{1}\to {M}_{O2}$ and ${O}_{1}\to {M}_{O2}$; 3b) ${A}_{1}\to {M}_{O2}$,${O}_{1}\to {M}_{O2}$ and $Y\to {M}_{O2}$; 4a)${O}_{2}\to {M}_{A2}$; and 4b) ${O}_{2}\to {M}_{A2}$ and $Y\to {M}_{A2}$. ^d^ Monte Carlo error less than 0.0035 for all estimates

**Fig. 6:**
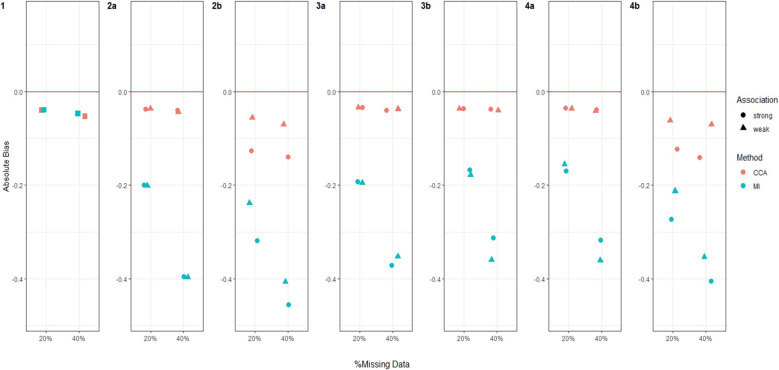
Bias in estimating stage 1 treatment effect ${\psi }_{10}$ under Treatment effect 5 simulation setting^a^. Footnotes: ^a^ True value of ${\psi }_{10}$ = 0.5 for Treatment effect 5 simulation setting when there is no treatment effect at stage 2 for half of the participants, but a relatively large effect for the other half. This simulation setting is similar to Treatment effect 3 but we assume that there is an even larger treatment effect at stage 2 for the other half. ^b^ Complete case analysis (CCA) and multiple imputation (MI) were used to handle missing data, where 20% or 40% had incomplete data under the seven missing data scenarios described in the Missingness in SMART designs section, see Fig. 2. ^c^ For weak association between the missing indicator and other variables (as described below) an OR of 1.6 was used; and for a strong association an OR of 3 was used. The other variables were: missing data scenario 2a) ${A}_{2}\to {M}_{Y}$; 2b)$Y\to {M}_{O2}$ and ${A}_{2}\to {M}_{Y}$; 3a) ${A}_{1}\to {M}_{O2}$ and ${O}_{1}\to {M}_{O2}$; 3b) ${A}_{1}\to {M}_{O2}$,${O}_{1}\to {M}_{O2}$ and $Y\to {M}_{O2}$; 4a)${O}_{2}\to {M}_{A2}$; and 4b) ${O}_{2}\to {M}_{A2}$ and $Y\to {M}_{A2}$. ^d^ Monte Carlo error less than 0.0035 for all estimates

#### Bias

For both CCA and MI, we observed close to zero bias under all missing data scenarios when there was no treatment effect at either stage 1 or 2 for any participants (Treatment effect 1, Fig. 3). When all participants had a relatively large treatment effect at stage 2 (Treatment effect 2, Fig. 4), we observed slightly more bias with MI than CCA under missing data scenarios 2b and 3a, albeit this bias was minimal. For missing data scenarios 2b and 3a, the bias was greater for stronger associations (~ threefold increase compared to weaker relationships of ~ 1.6-fold increase) between missing indicators ${O}_{2}$, ${A}_{2}$ and/or $Y$ and their predictors of missingness.

When the treatment effect at stage 2 varied between participants (Treatment effect 3, 4, 5), for example when half of the population had a very weak stage 2 treatment effect while the other half having a relatively large effect (Treatment effect 4, Fig. 5), MI showed greater bias than CCA. For these scenarios, we observed an increase in absolute bias for MI when the percentage of missing data increased from 20 to 40% but observed no change for CCA. For all simulation settings where treatment effect at stage 2 varied between participants, bias was not associated with the strength of associations between missing indicators and their predictors of missingness except for missing data scenario 2b (where ${O}_{2}$ missing dependent on $Y$ and $Y$ missing dependent on ${A}_{2}$) and 4b (where ${A}_{2}$ missing dependent on ${O}_{2}$ and $Y$) when half of the participants had no stage 2 treatment effect but the other half had a large stage 2 treatment effect (Treatment effect 5, Fig. 6), where there was greater bias for both missing data methods when the associations were stronger.

When estimating the stage 2 treatment effect, for both CCA and MI, we observed negligible bias for most missing data scenarios and simulation settings (Additional File 4: Tables S5-S9), except when the missingness in ${O}_{2}$ was dependent on a common cause between ${M}_{O2}$ and $Y$; and missingness in the stage 2 outcome data was dependent on ${A}_{2}$ (m-DAG 2a, Fig. 2), where both methods showed bias.

#### Empirical standard errors and Mean squared errors (MSE)

The empirical standard errors and MSE are shown in Additional file 3: Figures S2–S6. When there is a relatively large treatment effect at stage 2 for every participant (Treatment effect 2), we observed higher empirical standard errors for MI compared to CCA, under all missing data scenarios except for when stage 1 responder status and stage 2 outcome were missing not dependent on any variables (missing data scenario 1). For all other Treatment effect settings (e.g. no treatment effect at either stage 1 or 2, varying treatment effects at stage 2 between participants), the empirical standard errors were generally lower for MI compared to CCA under all missing data scenarios. As expected, empirical standard errors increased with the percentage of missingness. Stronger and weaker associations between the missing data indicators and their predictors of missingness showed similar empirical standard errors.

The MSE for CCA was similar across all treatment effect settings and missing data scenarios. When there was no treatment effect at either stage 1 or 2 for any participant, we observed similar MSE for both MI and CCA under all missing data scenarios (Additional file 3: Figure S2). For all other treatment effect settings (Additional file 3: Figures S3–S6), under all missing data scenarios except for missing data scenario 1, MI showed greater MSE compared to CCA which increased with the proportion of missingness. For missing data scenario 1, we observed similar MSE for both MI and CCA.

The bootstrap model-based standard errors produced similar values to the empirical-based standard errors (Additional file 4: Tables S2-S4). Lower coverage was observed with the treatment effect 4 simulation setting (Additional file 4: Table S4).

### Real data example

There were 479 participants that received interventions at both stages and 216 participants with outcome data available at stage 2 (55% missing in the outcome). For CCA, the point estimate of the stage 1 treatment effect, the estimated mean difference in number of attempts to quit smoking in participants using a web-based smoking cessation program compared to no program, was −0.147 (95% confidence interval: −0.752, 0.338). In comparison, when MI was used to handle the missing data the estimate of the stage 1 treatment effect was −0.190 (95% confidence interval: −0.617, 0.244). Both CCA and MI provided similar estimates.

## Discussion

In this simulation study, we evaluated the performance of CCA and MI for handling missing data when Q-learning is used to estimate the stage 1 treatment effect from the data of a typical two-stage SMART. We found that when stage 1 responder status and stage 2 outcome were missing not dependent on any variables (m-DAG 1, Fig. 2), we observed expected unbiased estimates for both CCA and MI across all simulation settings for the treatment effects. When there was no treatment effect at stage 1 or stage 2, both MI and CCA showed negligible absolute bias and similar empirical standard errors under all missing data scenarios. However, when the treatment effect at stage 2 varied between participants and data were missing dependent on other variables (for example, stage 1 responder status missing dependent on stage 1 treatment and baseline variables), MI showed greater bias which increased with the percentage missingness, while the bias for CCA remained minimal or negligible. When the treatment effect at stage 2 varied between participants, we observed lower or similar empirical standard errors for MI compared to CCA under all missing data scenarios. When all participants had a relatively large stage 2 treatment effect, we observed some, albeit minimal bias, with slightly greater bias for MI. The empirical standard errors were also higher for MI compared to CCA under all scenarios except for when data were missing not dependent on any variables. The stage 2 treatment effect was estimated via the parameters of the Q-learning model at stage 2 which is a standard linear regression model. We observed that under most missing data scenarios and simulation settings both CCA and MI showed negligible bias when estimating the stage 2 treatment effect.

In a case study of a smoking cessation study, we obtained similar estimates for both CCA and MI.

Many studies have shown that in standard, single stage trials, when outcome data are missing only dependent on observed data, MI shows an advantage over a CCA in handling missing data as it is able to capture the uncertainty in the missing data [9, 21]. However, for two stage trials such as SMARTs when the analysis is performed in stages using a backward induction approach (Q-learning), we found that MI induced bias while CCA showed negligible bias. When the treatment effect at stage 2 varied between participants, MI failed to capture these varied treatment effects. The backward induction nature of Q-learning caused difficulty in selecting an appropriate imputation model for MI, especially in how to appropriately account for the pseudo-outcome derived in the first regression step and included in the second regression step. Compatibility of the target analysis and imputation model is known to be important when MI is used to handle missing data [11] and from our simulation study it appears likely that Q-learning causes issues with this compatibility. Therefore, these issues may have contributed to the bias seen in MI.

Only a single study has investigated methods to handle missing data in SMARTs previously. Shortreed et al. [10] proposed an MI strategy to overcome challenges to handle missing data in SMARTs and applied this strategy on data from a case study, the Clinical Antipsychotic Trials of Intervention and Effectiveness (CATIE) study. They used weighted regression to estimate the mean response for each treatment regime, where weights were used to adjust for the probability of randomisation (similar to inverse probability weighting (IPW) used to adjust for confounding). In Shortreed et al.’s paper, CCA produced lower and more variable estimates compared to MI. In our study, we evaluated how well MI performed when Q-learning was used to find the optimal DTR and looked at how the varying treatment effects at each stage affected the performance of CCA and MI. Our simulation results showed that CCA produced less biased estimates compared to MI. Despite these findings from simulation studies, when applying CCA and MI to real data examples (PQ study and FF study) both missing data methods showed similar results.

Our paper considered four missing data scenarios, depicted using m-DAGs [6], that we might observe in a simple two-stage two-treatment SMART. These m-DAGs allow us to clearly outline the causal relationships between variables and their missingness and are critical for complex settings such as SMARTs given the multiple stages. Using m-DAGs to understand the missing data assumptions which describe how the data become missing is important as they help us choose appropriate methods to handle the missing data. However, our results are restricted to the simulation scenarios proposed [14] and the Q-learning analysis method [5]. Further research is needed to emulate this study for different targets of analysis (e.g. the mean of embedded DTRs, calculating the mean outcome if all participants follow one of the DTRs), estimation methods (e.g. such as IPW, G-estimation and weighted regression [3]), different missing data methods (e.g. IPW) and missing data causal diagrams including auxiliary variables or when missingness in the outcome also depends on the outcome values itself. It would also be useful to conduct a theoretical evaluation of the analytic approaches considered here to further understand their performance in the context of SMARTs.

## Conclusions

In conclusion, when handling missing data in a two-stage SMART, our results have shown that CCA is less biased compared to MI when estimating the stage 1 treatment effect. Since SMART designs have multiple stages, we recommend using m-DAGs to outline the missing data assumptions used in the analysis.

## Supplementary Information

Supplementary Material 1.

Supplementary Material 2.

Supplementary Material 3.

Supplementary Material 4.

## Data Availability

The statistical computing code for this simulation study is available on GitHub https://github.com/jessicaxu3205/SMART_missing. Data from the Project Quit (PQ) study and the Forever Free (FF) study were made available by the Principal Investigator (Victor J. Strecher, University of Michigan, US) and Bibhas Chakraborty (Duke NUS, Singapore) and are available from the authors upon reasonable request and with permission of Victor J. Strecher and Bibhas Chakraborty.

## Abbreviations

CATIE: Clinical Antipsychotic Trials of Intervention and Effectiveness
CCA: Complete Case Analysis
DTR: Dynamic Treatment Regimen
FF: Forever Free
IPW: Inverse-probability Weighting
MI: Multiple Imputation
m-DAG: Missing Directed Acyclic Graph
MSE: Mean Squared Error
PQ: Project Quit
RCT: Randomised Controlled Trial
SMART: Sequential Multiple Assignment Randomised Trial
