# Supplementary material for: Optimising dynamic treatment regimens using sequential multiple assignment randomised trials data with missing data
Source: BMC Med Res Methodol. 2025 Jul 1;25:162. doi: 10.1186/s12874-025-02595-1 (PMC12211643; doi:10.1186/s12874-025-02595-1)
Supplement: Supplementary file 1 — Supplementary Material 1. [file 12874_2025_2595_MOESM1_ESM.docx]

**Additional file 1**

Table S1: Parameter values used in the logistic regression models when generating missing data under different scenarios.

| **Variables with  missing data** | **Missing data mechanism** | | **Association  [**$\boldsymbol{\alpha}_{\boldsymbol{1}}\boldsymbol{=}\boldsymbol{\alpha}_{\boldsymbol{2}}\boldsymbol{=}\boldsymbol{\alpha}_{\boldsymbol{3}}\boldsymbol{=}\text{log}\boldsymbol{(}\text{Odds}\text{ }\text{ratio}\boldsymbol{)]}$ | | **Intercepts^a^  (**$\boldsymbol{\alpha}_{\boldsymbol{0}}$**)** | |
| --- | --- | --- | --- | --- | --- | --- |
|  |  |  |  |  | **20% missingness** | **40% missingness** |
| **Missing data scenario 1** | |  |  |  |  |  |
| $O_{2}$ | Random |  | - | | - | - |
| $Y$ | Random |  | - | | - | - |
| **Missing data scenario 2a** | | | |  |  |  |
| $O_{2}$ | Random |  | - | | - | - |
| $A_{2}$ | If $O_{2}$ is missing |  | - | | - | - |
| $Y$ | $\text{logit}[P (M_{Y} = 1)] = \alpha_{0} + \alpha_{1}[A_{2} = 1]$ and if $O_{2}$ and/or $A_{2}$ is missing | | Weak [$\alpha_{1}=\text{log}(1.6)$] | | -5 | -4.2 |
|  |  |  | Strong [$\alpha_{1}=\text{log}(3)$] | | -5 | -4.2 |
| **Missing data scenario 2b** | | | |  |  |  |
| $O_{2}$ | $\text{logit}[P (M_{O2} = 1)] = \alpha_{0} + \alpha_{1}Y$ |  | Weak [$\alpha_{1}=\text{log}(1.6)$] | | -1.5 | -0.5 |
|  |  |  | Strong [$\alpha_{1}=\text{log}(3)$] | | -2 | -0.7 |
| $A_{2}$ | If $O_{2}$ is missing |  | - | | - | - |
| $Y$ | $\text{logit}[P (M_{Y} = 1)] = \alpha_{0} + \alpha_{1}[A_{2} = 1]$ and if $O_{2}$ and/or $A_{2}$ is missing | | Weak [$\alpha_{1}=\text{log}(1.6)$] | | -4.5 | -3 |
|  |  |  | Strong [$\alpha_{1}=\text{log}(3)$] | | -4.2 | -4.5 |
| **Missing data scenario 3a** | | |  |  |  |  |
| $O_{2}$ | $\text{logit}[P (M_{02} = 1)] = \alpha_{0} + \alpha_{1}[O_{1} = 1] + \alpha_{2}[A_{1} = 1]$ | | Weak [$\alpha_{1}=\alpha_{2}=\text{log}(1.6)$] | | -1.5 | -0.6 |
|  |  | | Strong [$\alpha_{1}=\alpha_{2}=\text{log}(3)$] | | -2.2 | -1 |
| $A_{2}$ | If $O_{2}$ is missing |  | - | | - | - |
| $Y$ | If $O_{2}$ and/or $A_{2}$ is missing |  | - | | - | - |
| **Missing data scenario 3b** | | |  |  |  |  |
| $O_{2}$ | $logit[P (M_{02} = 1)] = \alpha_{0} + \alpha_{1}[O_{1} = 1] + \alpha_{2}[A_{1} = 1] + \alpha_{3}Y$ | | Weak [$\alpha_{1}=\alpha_{2}=\alpha_{3}=\text{log}(1.6)$ | | -1.6 | -0.6 |
|  |  | | Strong [$\alpha_{1}=\alpha_{2}=\alpha_{3}=\text{log}(3)$] | | -2 | -0.9 |
| $A_{2}$ | If $O_{2}$ is missing |  | - | | - | - |
| $Y$ | If $O_{2}$ and/or $A_{2}$ is missing |  | - | | - | - |
| **Missing data scenario 4a** | | |  |  |  |  |
| $A_{2}$ | $logit[P (M_{A2} = 1)] = \alpha_{0} + \alpha_{1}[O_{2} = 1]$ | | Weak [$\alpha_{1}=\text{log}(1.6)$] | | -1.8 | -0.6 |
|  |  | | Strong [$\alpha_{1}=\text{log}(3)$] | | -2.2 | -1.2 |
| $Y$ | If $A_{2}$ is missing |  | - | | - | - |
| **Missing data scenario 4b** | | |  |  |  |  |
| $A_{2}$ | $logit[P (M_{A2} = 1)] = \alpha_{0} + \alpha_{1}[O_{2} = 1] + \alpha_{2}Y$ | | Weak [$\alpha_{1}=\alpha_{2}=\text{log}(1.6)$] | | -1.8 | -0.8 |
|  |  | | Strong [$\alpha_{1}=\alpha_{2}=\text{log}(3)$] | | -2.7 | -1.4 |
| $Y$ | If $A_{2}$ is missing |  | - | | - | - |

Footnotes: ^a^ The intercepts ($\alpha_{0}$) were chosen by iteration to achieve the required percentage of missingness.
