# Supplementary material for: Optimising dynamic treatment regimens using sequential multiple assignment randomised trials data with missing data
Source: BMC Med Res Methodol. 2025 Jul 1;25:162. doi: 10.1186/s12874-025-02595-1 (PMC12211643; doi:10.1186/s12874-025-02595-1)
Supplement: Supplementary file 2 — Supplementary Material 2. [file 12874_2025_2595_MOESM2_ESM.docx]

**Additional file 2**

**Non-regularity**

Non-regularity occurs when there is no unique optimal treatment at stages after stage 1 for at least some participants. This means that these participants would have no treatment effect at stage 2. For this reason, we focus on estimation of the stage 1 treatment effect parameter $\psi_{1}$.

Non-regularity (as described by Chakraborty et al. [14]) occurs when the asymptotic distribution of the Q-learning estimator does not converge uniformly to a normal distribution. This implies that, as the sample size gets larger towards infinity, the distribution of the estimator $\psi_{1}$ is not the same for all parameter values and doesn’t converge to a normal distribution.

For Q-learning, non-regularity arises from the non-smooth absolute function ($\left| \hat{\psi}_{2}^{T}H_{2i} \right|$) used to construct the stage 1 pseudo-outcome (see Equation 7 from main text), which includes the $\psi_{2}$ parameters that are associated with stage 2 treatment effects. Since absolute functions are non-differentiable at 0, when the probability of $\hat{\psi}_{2}^{T}H_{2i}=0$ is 0, the asymptotic distribution of the Q-learning estimator is normal and when the probability of $\hat{\psi}_{2}^{T}H_{2i}=0$ is greater than 0, it is non-normal. Therefore, assumptions on which large-sample theory rely on are violated, which may lead to poor confidence interval coverage and biased inferences. Non-regularity can be a problem when Q-learning is used to estimate treatment effect parameters for data collected at multiple stages as there would be no unique optimal treatment in the presence of non-regularity.

To understand how Q-learning parameters are affected by the different non-regularity settings, Chakraborty et al. [14] proposed 6 simulation settings as follows:

- Setting 1: (fully non-regular) there is no treatment effect on $Y$ in either stage 1 or 2 for any participant.
- Setting 2: (regular, but close to non-regular) there is a very weak treatment effect on $Y$ at stage 2 for any participant.
- Setting 3: (non-regular) there is no treatment effect on $Y$ at stage 2 for half of the participants, but a relatively large effect for the other half.
- Setting 4: (regular, but close to non-regular) there is a very weak treatment effect on $Y$ at stage 2 for half of the participants, but a relatively large effect for the other half.
- Setting 5: (non-regular) there is no treatment effect on $Y$ at stage 2 for a quarter of the participants, but a relatively large effect for the others.
- Setting 6: (fully regular) there is a relatively large treatment effect on $Y$ at stage 2 for every participant.
