# Supplementary material for: Optimising dynamic treatment regimens using sequential multiple assignment randomised trials data with missing data
Source: BMC Med Res Methodol. 2025 Jul 1;25:162. doi: 10.1186/s12874-025-02595-1 (PMC12211643; doi:10.1186/s12874-025-02595-1)
Supplement: Supplementary file 3 — Supplementary Material 3. [file 12874_2025_2595_MOESM3_ESM.docx]

**Additional file 3**

For all figures below the seven missing data scenarios (see Figure 2 from main text) are as follows, where $O_{1}$ = baseline characteristic; $A_{1}$= stage 1 treatment; $O_{2}$= stage 1 responder status; $A_{2}$= stage 2 treatment; $Y$= stage 2 outcome:

- Missing data scenario 1: $O_{2}$ and $Y$ are missing not dependent on other variables.
- Missing data scenario 2a: $O_{2}$ is missing not dependent on other variables and $Y$ missing dependent on $A_{2}$.
- Missing data scenario 2b: $O_{2}$ missing dependent on a common cause between $M_{O2}$ and $Y$ which induces an association between $M_{O2}$ and $Y$; $Y$ missing dependent on $A_{2}$ and if $O_{2}$ is missing.
- Missing data scenario 3a: $O_{2}$ missing dependent on $A_{1}$ and $O_{1}$; $Y$ missing if $O_{2}$ missing.
- Missing data scenario 3b: $O_{2}$ missing dependent on $A_{1}$, $O_{1}$ and a common cause between $M_{O2}$ and $Y$ which induces an association between $M_{O2}$ and $Y$; $Y$ missing if $O_{2}$ missing.
- Missing data scenario 4a: $A_{2}$ missing dependent on $O_{2}$ and $Y$ missing if $A_{2}$ missing.
- Missing data scenario 4b: $A_{2}$ missing dependent on $O_{2}$ and $Y$ (assuming a common cause of missingness in $A_{2}$ and $Y$); $Y$ missing if $A_{2}$ missing.

Figure S1: Bias in estimating stage 1 treatment effect $\psi_{10}$ under Treatment effect 3 simulation setting^a^.


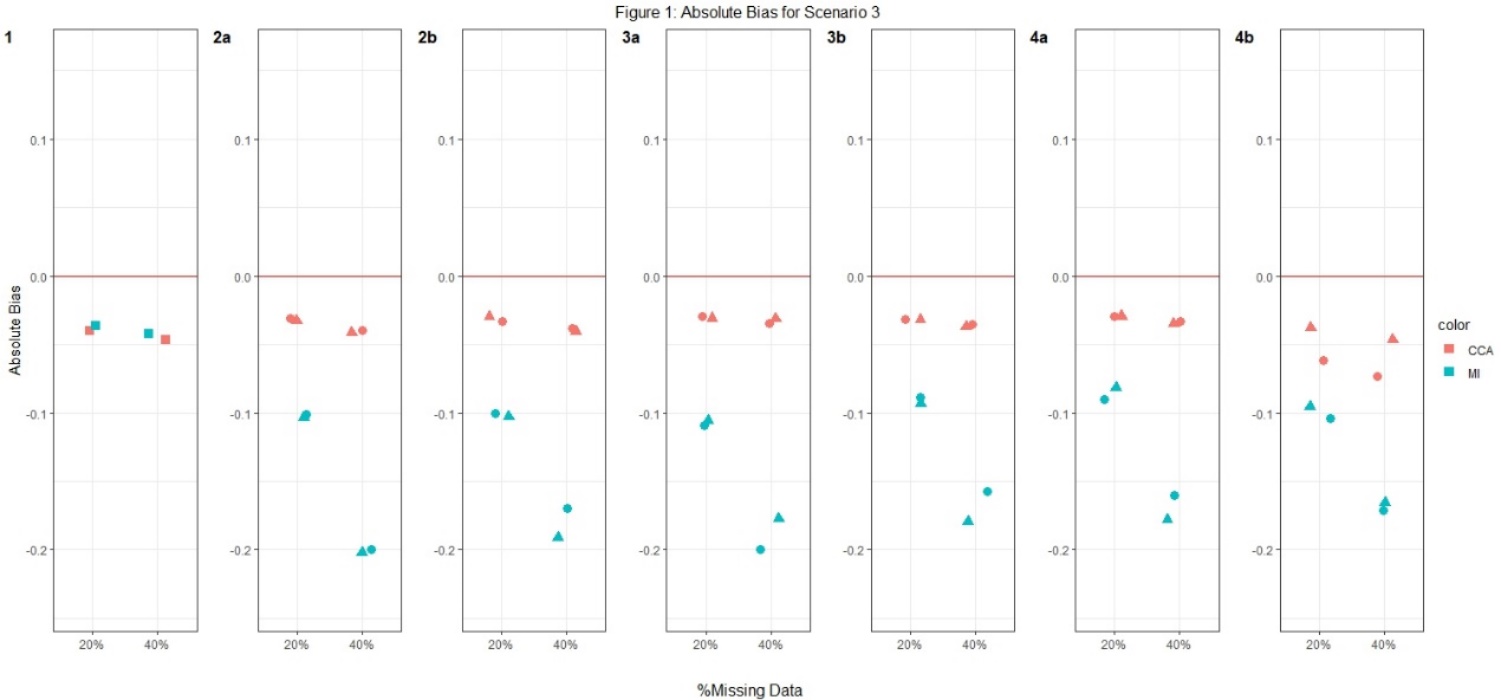

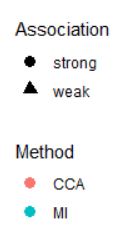


Footnotes: : ^a^ True value of $\psi_{10}$ = 0 for Treatment effect 3 simulation setting when there is no treatment effect at stage 2 for half of the participants, but a relatively large effect for the other half.

^b^ Complete case analysis (CCA) and multiple imputation (MI) were used to handle missing data, where 20% or 40% had incomplete data under the seven missing data scenarios described in the Missingness in SMART designs section, see Figure 2.

^c^ For weak association between the missing indicator and other variables (as described below) an odds ratio (OR) of 1.6 was used; and for a strong association an OR of 3 was used. The other variables were: missing data scenario 2a) $A_{2}\to M_{Y}$; 2b)$Y\to M_{O2}$ and $A_{2}\to M_{Y}$; 3a) $A_{1}\to M_{O2}$ and $O_{1}\to M_{O2}$; 3b) $A_{1}\to M_{O2}$,$O_{1}\to M_{O2}$ and $Y\to M_{O2}$; 4a)$O_{2}\to M_{A2}$; and 4b) $O_{2}\to M_{A2}$ and $Y\to M_{A2}$.

^d^ Monte Carlo error less than 0.0035 for all estimates.

Figure S2: Empirical standard errors and mean squared error for $\psi_{10}$ under Treatment effect 1 simulation setting^a^.


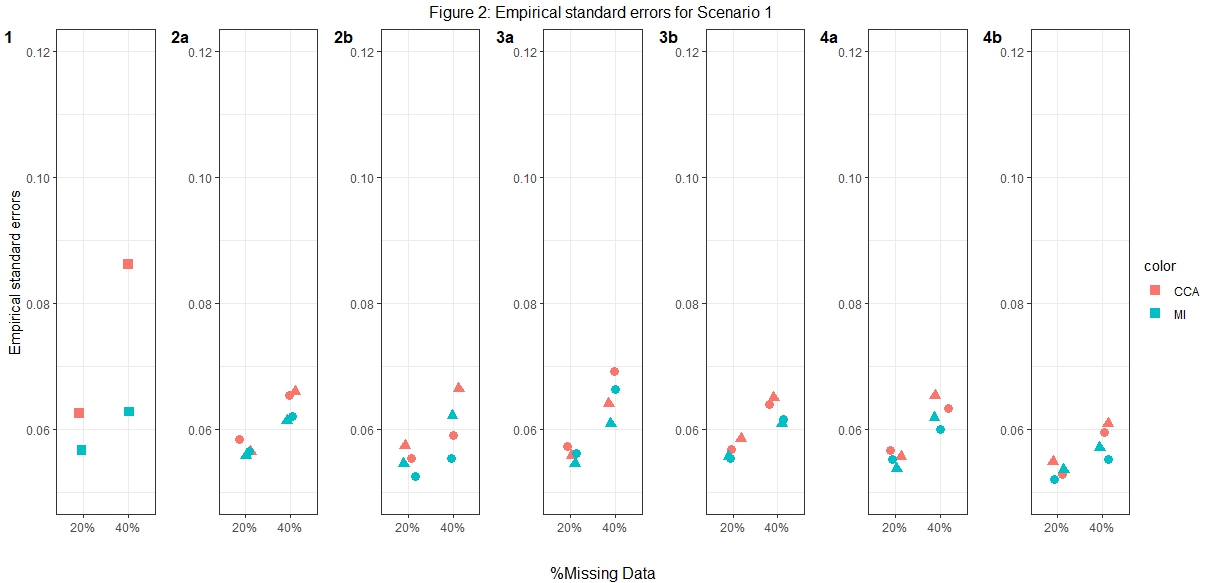

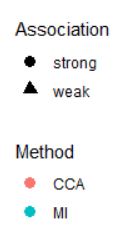


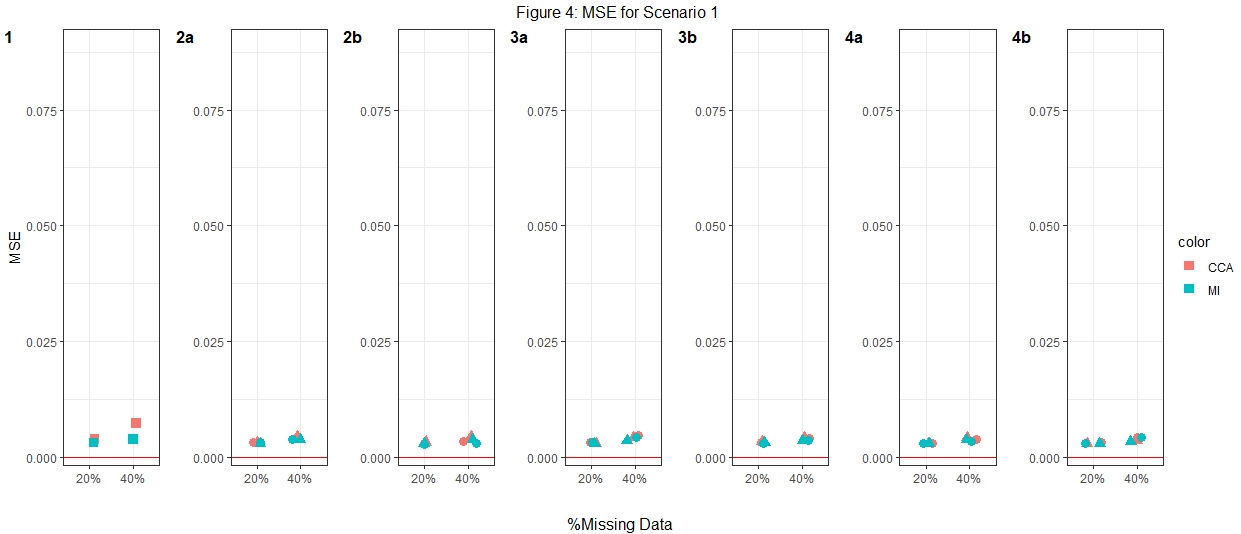


Footnotes: ^a^ True value of stage 1 treatment effect $\psi_{10}$ = 0 for Treatment effect 1 simulation setting when there is no treatment effect at either stage 1 or 2 for any participant.

^b^ MSE= mean square error

^c^ Complete case analysis (CCA) and multiple imputation (MI) were used to handle missing data, where 20% or 40% had incomplete data under the seven missing data scenarios described in the Missingness in SMART designs section, see Figure 2.

^d^ For weak association between the missing indicator and other variables (as described below) an odds ratio (OR) of 1.6 was used; and for a strong association an OR of 3 was used. The other variables were: missing data scenario 2a) $A_{2}\to M_{Y}$; 2b)$Y\to M_{O2}$ and $A_{2}\to M_{Y}$; 3a) $A_{1}\to M_{O2}$ and $O_{1}\to M_{O2}$; 3b) $A_{1}\to M_{O2}$,$O_{1}\to M_{O2}$ and $Y\to M_{O2}$; 4a)$O_{2}\to M_{A2}$; and 4b) $O_{2}\to M_{A2}$ and $Y\to M_{A2}$.

^e^ Monte Carlo error less than 0. 0025 for all estimates.

Figure S3: Empirical standard errors and mean squared error for $\psi_{10}$ under Treatment effect 2 simulation setting^a^.


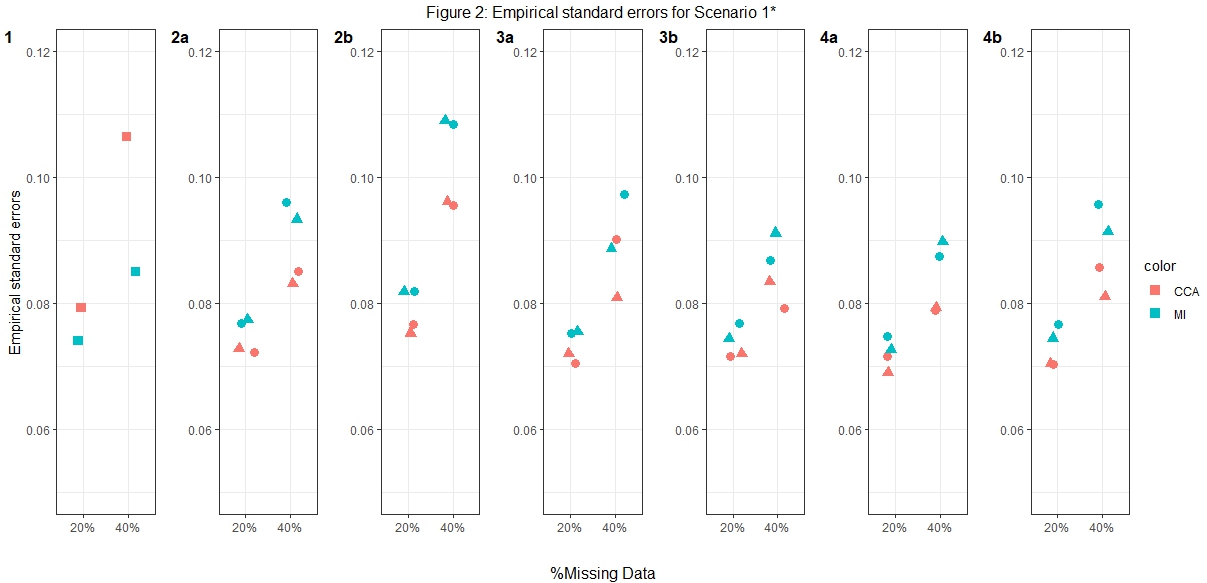

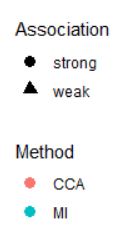


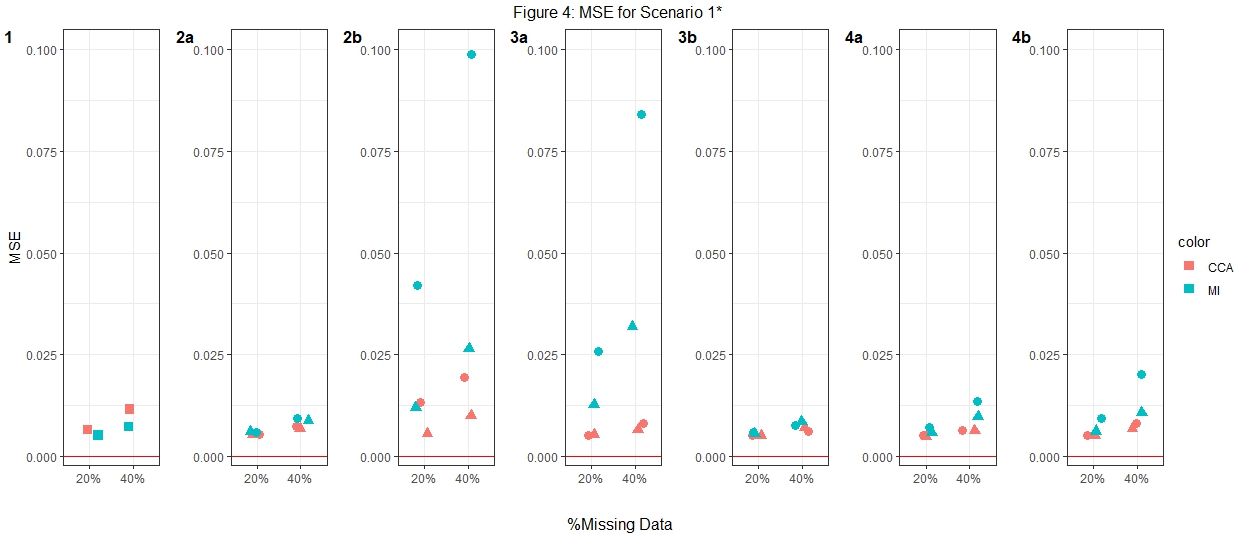


Footnotes: ^a^ True value of stage 1 treatment effect $\psi_{10}$ = -0.5 for Treatment effect 2 simulation setting when there is a relatively large treatment effect at stage 2 for every participant.

^b^ MSE= mean square error

^c^ Complete case analysis (CCA) and multiple imputation (MI) were used to handle missing data, where 20% or 40% had incomplete data under the seven missing data scenarios described in the Missingness in SMART designs section, see Figure 2.

^d^ For weak association between the missing indicator and other variables (as described below) an odds ratio (OR) of 1.6 was used; and for a strong association an OR of 3 was used. The other variables were: missing data scenario 2a) $A_{2}\to M_{Y}$; 2b)$Y\to M_{O2}$ and $A_{2}\to M_{Y}$; 3a) $A_{1}\to M_{O2}$ and $O_{1}\to M_{O2}$; 3b) $A_{1}\to M_{O2}$,$O_{1}\to M_{O2}$ and $Y\to M_{O2}$; 4a)$O_{2}\to M_{A2}$; and 4b) $O_{2}\to M_{A2}$ and $Y\to M_{A2}$.

^e^ Monte Carlo error less than 0. 0025 for all estimates.

Figure S4: Empirical standard errors and mean squared error for $\psi_{10}$ under Treatment effect 3 simulation setting^a^.


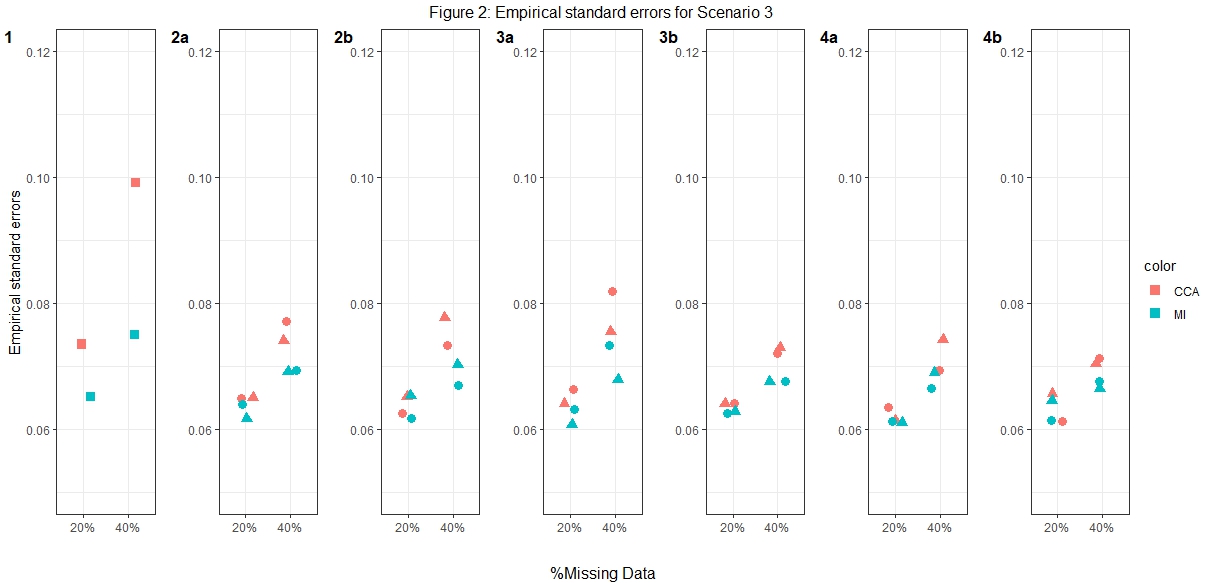

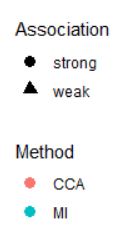


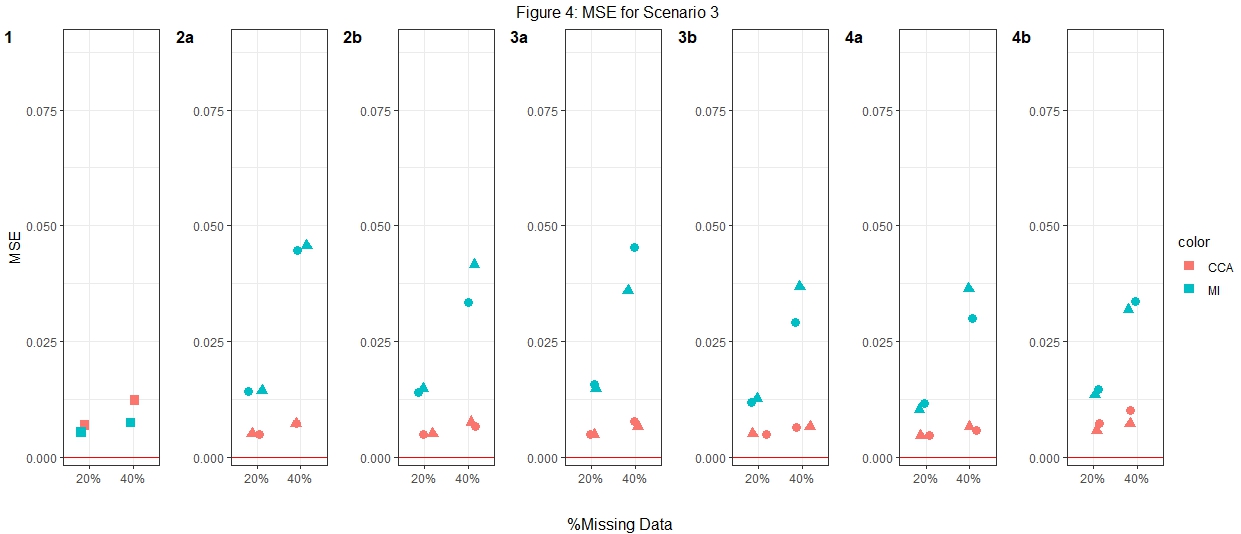


Footnotes: ^a^  True value of stage 1 treatment effect $\psi_{10}$ = 0 for Treatment effect 3 simulation setting when there is no treatment effect at stage 2 for half of the participants, but a relatively large effect for the other half.

^b^ MSE= mean square error

^c^ Complete case analysis (CCA) and multiple imputation (MI) were used to handle missing data, where 20% or 40% had incomplete data under the seven missing data scenarios described in the Missingness in SMART designs section, see Figure 2.

^d^ For weak association between the missing indicator and other variables (as described below) an OR of 1.6 was used; and for a strong association an OR of 3 was used. The other variables were: missing data scenario 2a) $A_{2}\to M_{Y}$; 2b)$Y\to M_{O2}$ and $A_{2}\to M_{Y}$; 3a) $A_{1}\to M_{O2}$ and $O_{1}\to M_{O2}$; 3b) $A_{1}\to M_{O2}$,$O_{1}\to M_{O2}$ and $Y\to M_{O2}$; 4a)$O_{2}\to M_{A2}$; and 4b) $O_{2}\to M_{A2}$ and $Y\to M_{A2}$.

^e^ Monte Carlo error less than 0. 0025 for all estimates.

Figure S5: Empirical standard errors and mean squared error for $\psi_{10}$ under Treatment effect 4 simulation setting^a^.


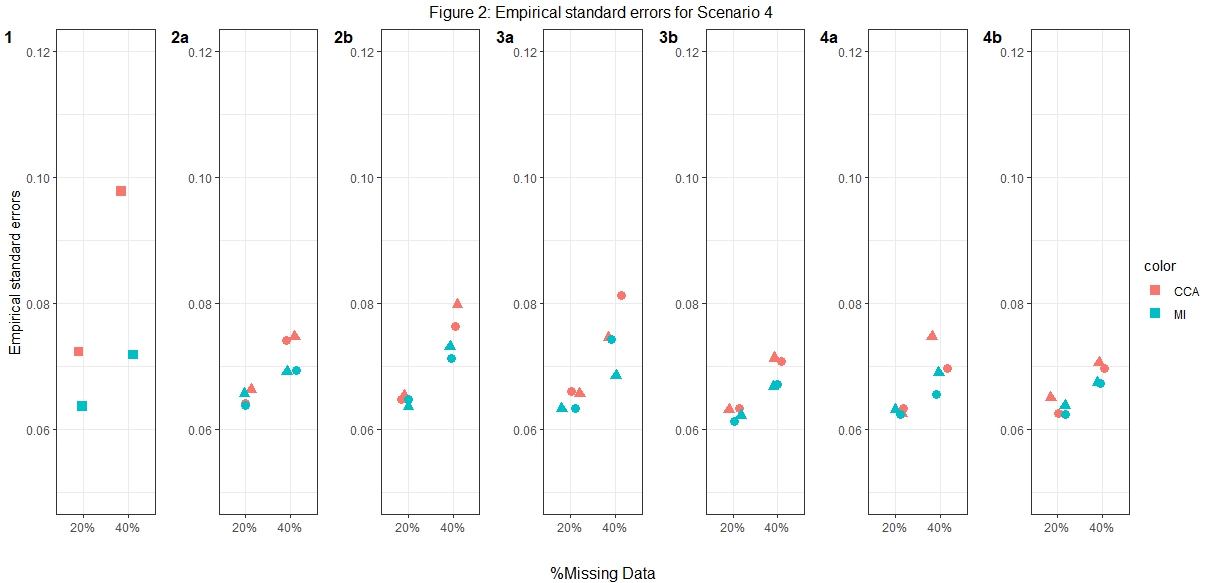

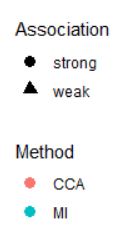


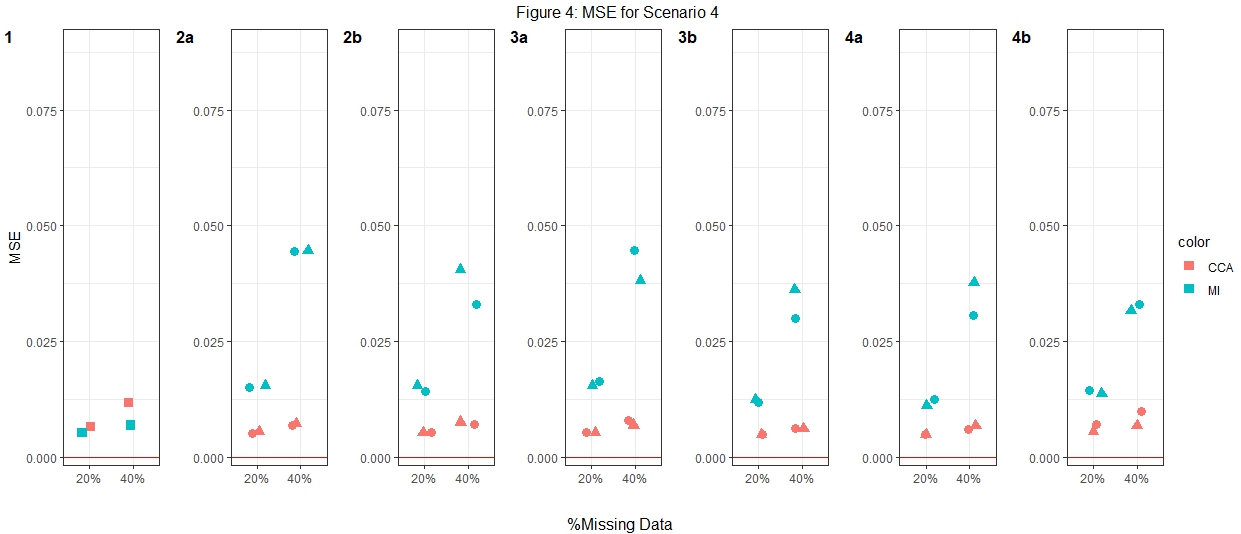


Footnotes: ^a^ True value of stage 1 treatment effect $\psi_{10}$ = -0.01 for Treatment effect 4 simulation setting when there is a very weak treatment effect at stage 2 for half of the participants, but a relatively large effect for the other half.

^b^ MSE= mean square error

^c^ Complete case analysis (CCA) and multiple imputation (MI) were used to handle missing data, where 20% or 40% had incomplete data under the seven missing data scenarios described in the Missingness in SMART designs section, see Figure 2.

^d^ For weak association between the missing indicator and other variables (as described below) an odds ratio (OR) of 1.6 was used; and for a strong association an OR of 3 was used. The other variables were: missing data scenario 2a) $A_{2}\to M_{Y}$; 2b)$Y\to M_{O2}$ and $A_{2}\to M_{Y}$; 3a) $A_{1}\to M_{O2}$ and $O_{1}\to M_{O2}$; 3b) $A_{1}\to M_{O2}$,$O_{1}\to M_{O2}$ and $Y\to M_{O2}$; 4a)$O_{2}\to M_{A2}$; and 4b) $O_{2}\to M_{A2}$ and $Y\to M_{A2}$.

^e^ Monte Carlo error less than 0. 0025 for all estimates.

Figure S6: Empirical standard errors and mean squared error for $\psi_{10}$ under Treatment effect 5 simulation setting^a^.


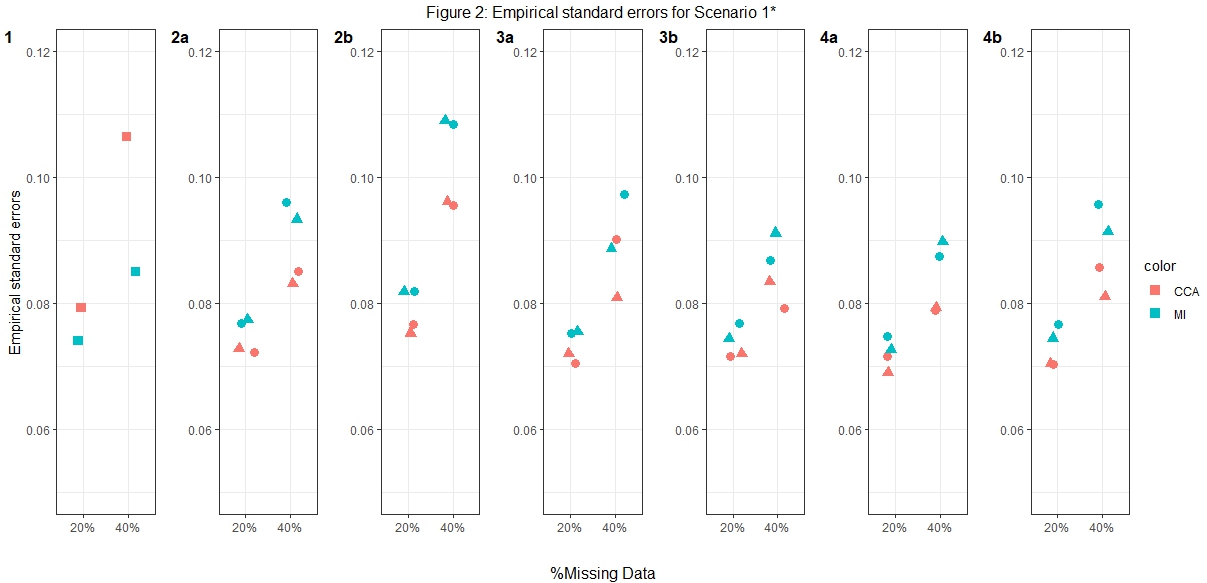

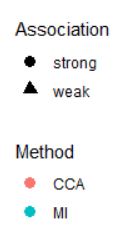


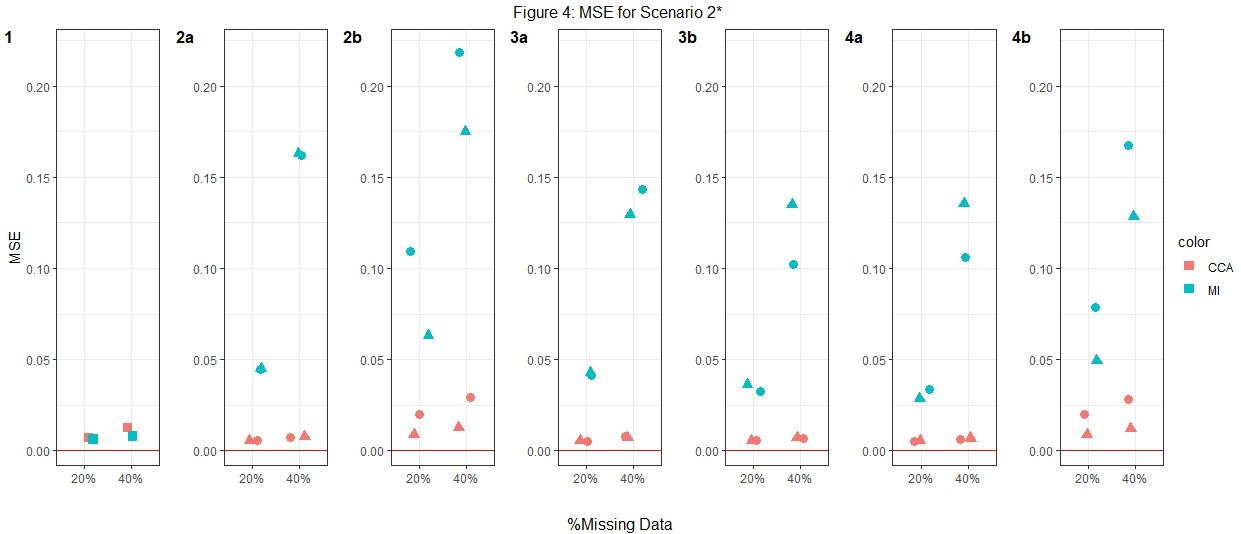


Footnotes: ^a^ True value of stage 1 treatment effect $\psi_{10}$ = 0.5 for Treatment effect 5 simulation setting when there is no treatment effect at stage 2 for half of the participants, but a relatively large effect for the other half. This simulation setting is similar to Treatment effect 3 but we assume that there is an even larger treatment effect at stage 2 for the other half.

^b^ MSE= mean square error

^c^ Complete case analysis (CCA) and multiple imputation (MI) were used to handle missing data, where 20% or 40% had incomplete data under the seven missing data scenarios described in the Missingness in SMART designs section, see Figure 2.

^d^ For weak association between the missing indicator and other variables (as described below) an odds ratio (OR) of 1.6 was used; and for a strong association an OR of 3 was used. The other variables were: missing data scenario 2a) $A_{2}\to M_{Y}$; 2b)$Y\to M_{O2}$ and $A_{2}\to M_{Y}$; 3a) $A_{1}\to M_{O2}$ and $O_{1}\to M_{O2}$; 3b) $A_{1}\to M_{O2}$,$O_{1}\to M_{O2}$ and $Y\to M_{O2}$; 4a)$O_{2}\to M_{A2}$; and 4b) $O_{2}\to M_{A2}$ and $Y\to M_{A2}$.

^e^ Monte Carlo error less than 0. 0025 for all estimates.
