# Supplementary material for: Optimising dynamic treatment regimens using sequential multiple assignment randomised trials data with missing data
Source: BMC Med Res Methodol. 2025 Jul 1;25:162. doi: 10.1186/s12874-025-02595-1 (PMC12211643; doi:10.1186/s12874-025-02595-1)
Supplement: Supplementary file 4 — Supplementary Material 4. [file 12874_2025_2595_MOESM4_ESM.docx]

**Additional file 4**

**Table S2: Summary statistics for stage 1 treatment effect estimate for Treatment effect 1 simulation setting. (True value=0). Note due to long computational time we have selected to present 3 treatment effect simulation settings and of those 2 missing data scenarios for the MI estimates.**

|  | Complete Case Analysis | | | | | Multiple Imputation | | | | |
| --- | --- | --- | --- | --- | --- | --- | --- | --- | --- | --- |
| Missing data scenarios | **Bias** | **Empirical S.E** | **Bootstrap Model-based S.E** | **MSE** | **Coverage** | **Bias** | **Empirical S.E** | **Bootstrap Model-based S.E** | **MSE** | **Coverage** |
|  | **m-DAG 1:** $\boldsymbol{O}_{\boldsymbol{2}}$ **and** $\boldsymbol{Y}$ **are missing not dependent on other variables** | | | | | | | | | |
| 20% missingness | -0.0002 | 0.0627 | 0.0664 | 0.0039 | 0.967 | 0.0029 | 0.0546 | 0.0660 | 0.0030 | 0.967 |
| 40% missingness | -0.0001 | 0.0863 | 0.0890 | 0.0075 | 0.960 | ~~-~~ | - | - | - | - |
|  | **m-DAG 2a:** $\boldsymbol{O}_{\boldsymbol{2}}$**is missing not dependent on other variables and** $\boldsymbol{Y}$ **missing dependent on** $\boldsymbol{A}_{\boldsymbol{2}}$ | | | | | | | | | |
| 20% missingness + weak association | 0.0033 | 0.0566 | 0.0595 | 0.0032 | 0.968 | 0.0031 | 0.0556 | 0.0594 | 0.0031 | 0.972 |
| 20% missingness + strong association | 0.0024 | 0.0556 | 0.0597 | 0.0031 | 0.968 | ~~-~~ | - | - | - | - |
| 40% missingness + weak association | 0.0029 | 0.0651 | 0.0694 | 0.0042 | 0.963 | ~~-~~ | - | - | - | - |
| 40% missingness + strong association | 0.0008 | 0.0647 | 0.0695 | 0.0042 | 0.965 | ~~-~~ | - | - | - | - |
|  | **m-DAG 3a:** $\boldsymbol{O}_{\boldsymbol{2}}$ **missing dependent on** $\boldsymbol{A}_{\boldsymbol{1}}$ **and** $\boldsymbol{O}_{\boldsymbol{1}}$**;** $\boldsymbol{Y}$ **missing if** $\boldsymbol{O}_{\boldsymbol{2}}$ **missing.** | | | | | | | | | |
| 20% missingness + weak association | 0.0061 | 0.0548 | 0.0593 | 0.0030 | 0.968 | ~~-~~ | - | - | - | - |
| 20% missingness + strong association | 0.0067 | 0.0557 | 0.0594 | 0.0031 | 0.958 | ~~-~~ | - | - | - | - |
| 40% missingness + weak association | 0.0074 | 0.0630 | 0.0674 | 0.0040 | 0.971 | ~~-~~ | - | - | - | - |
| 40% missingness + strong association | 0.0062 | 0.0687 | 0.0738 | 0.0048 | 0.955 | ~~-~~ | - | - | - | - |
|  | **m-DAG 4a:** $\boldsymbol{A}_{\boldsymbol{2}}$ **missing dependent on** $\boldsymbol{O}_{\boldsymbol{2}}$ **and** $\boldsymbol{Y}$ **missing if** $\boldsymbol{A}_{\boldsymbol{2}}$**missing.** | | | | | | | | | |
| 20% missingness + weak association | 0.0057 | 0.0538 | 0.0573 | 0.0029 | 0.961 | ~~-~~ | - | - | - | - |
| 20% missingness + strong association | 0.0045 | 0.0544 | 0.0579 | 0.0030 | 0.968 | ~~-~~ | - | - | - | - |
| 40% missingness + weak association | 0.0033 | 0.0623 | 0.0664 | 0.0039 | 0.962 | ~~-~~ | - | - | - | - |
| 40% missingness + strong association | 0.0084 | 0.0596 | 0.0650 | 0.0036 | 0.970 | ~~-~~ | - | - | - | - |

Footnotes: ^a^ Treatment effect 1 simulation setting when there is no treatment effect at either stage 1 or 2 for any participant.

^b^$O_{1}$= basic characteristic; $A_{1}$= stage 1 treatment; $O_{2}$= stage 1 responder status; $A_{2}$= stage 2 treatment; $Y$ = stage 2 outcome

^c^ For weak association between the missing indicator and other variables (as described below) an OR of 1.6 was used; and for a strong association an OR of 3 was used. The other variables were: missing data scenario 2a) $A_{2}$→$M_{Y}$ ; 3a) $A_{1}$→$M_{O2}$ and $O_{1}$→$M_{O2}$; and 4a) $O_{2}$→$M_{A2}$.

^d^ 200 bootstraps were used to obtain bootstrap model-based SEs and coverage.

**Table S3: Summary statistics for stage 1 treatment effect estimate for Treatment effect 2 simulation setting. (True value=-0.5) Note due to long computational time we have selected to present 3 treatment effect simulation settings and of those 2 missing data scenarios for the MI estimates.**

|  | Complete Case Analysis | | | | | Multiple Imputation | | | | |
| --- | --- | --- | --- | --- | --- | --- | --- | --- | --- | --- |
| Missing data scenarios | **Bias** | **Empirical S.E** | **Bootstrap Model-based S.E** | **MSE** | **Coverage** | **Bias** | **Empirical S.E** | **Bootstrap Model-based S.E** | **MSE** | **Coverage** |
|  | **m-DAG 1:** $\boldsymbol{O}_{\boldsymbol{2}}$ **and** $\boldsymbol{Y}$ **are missing not dependent on other variables** | | | | | | | | | |
| 20% missingness | -0.0008 | 0.0785 | 0.0797 | 0.0062 | 0.955 | 0.0004 | 0.0715 | 0.0713 | 0.0051 | 0.943 |
| 40% missingness | -0.0034 | 0.1110 | 0.1068 | 0.0123 | 0.924 | ~~-~~ | - | - | - | - |
|  | **m-DAG 2a:** $\boldsymbol{O}_{\boldsymbol{2}}$**is missing not dependent on other variables and** $\boldsymbol{Y}$ **missing dependent on** $\boldsymbol{A}_{\boldsymbol{2}}$ | | | | | | | | | |
| 20% missingness + weak association | -0.0014 | 0.0732 | 0.0713 | 0.0054 | 0.936 | -0.0025 | 0.0721 | 0.0713 | 0.0052 | 0.941 |
| 20% missingness + strong association | -0.0014 | 0.0691 | 0.0713 | 0.0048 | 0.936 | ~~-~~ | - | - | - | - |
| 40% missingness + weak association | -0.0007 | 0.0851 | 0.0828 | 0.0072 | 0.939 | ~~-~~ | - | - | - | - |
| 40% missingness + strong association | -0.003 | 0.0849 | 0.0832 | 0.0072 | 0.940 | ~~-~~ | - | - | - | - |
|  | **m-DAG 3a:** $\boldsymbol{O}_{\boldsymbol{2}}$ **missing dependent on** $\boldsymbol{A}_{\boldsymbol{1}}$ **and** $\boldsymbol{O}_{\boldsymbol{1}}$**;** $\boldsymbol{Y}$ **missing if** $\boldsymbol{O}_{\boldsymbol{2}}$ **missing.** | | | | | | | | | |
| 20% missingness + weak association | -0.0009 | 0.0725 | 0.0710 | 0.0053 | 0.928 | ~~-~~ | - | - | - | - |
| 20% missingness + strong association | -0.0017 | 0.0717 | 0.0715 | 0.0051 | 0.944 | ~~-~~ | - | - | - | - |
| 40% missingness + weak association | -0.0001 | 0.0808 | 0.0800 | 0.0065 | 0.936 | ~~-~~ | - | - | - | - |
| 40% missingness + strong association | -0.0057 | 0.0874 | 0.0863 | 0.0077 | 0.932 | ~~-~~ | - | - | - | - |
|  | **m-DAG 4a:** $\boldsymbol{A}_{\boldsymbol{2}}$ **missing dependent on** $\boldsymbol{O}_{\boldsymbol{2}}$ **and** $\boldsymbol{Y}$ **missing if** $\boldsymbol{A}_{\boldsymbol{2}}$**missing.** | | | | | | | | | |
| 20% missingness + weak association | -0.0006 | 0.0689 | 0.0691 | 0.0047 | 0.936 | ~~-~~ | - | - | - | - |
| 20% missingness + strong association | -0.0019 | 0.0713 | 0.0697 | 0.0051 | 0.940 | ~~-~~ | - | - | - | - |
| 40% missingness + weak association | -0.0046 | 0.0799 | 0.0798 | 0.0064 | 0.932 | ~~-~~ | - | - | - | - |
| 40% missingness + strong association | -0.0039 | 0.0756 | 0.0770 | 0.0057 | 0.935 | ~~-~~ | - | - | - | - |

Footnotes: ^a^ Treatment effect 2 simulation setting when there is a relatively large treatment effect at stage 2 for every participant.

^b^$O_{1}$= basic characteristic; $A_{1}$= stage 1 treatment; $O_{2}$= stage 1 responder status; $A_{2}$= stage 2 treatment; $Y$ = stage 2 outcome

^c^ For weak association between the missing indicator and other variables (as described below) an OR of 1.6 was used; and for a strong association an OR of 3 was used. The other variables were: missing data scenario 2a) $A_{2}$→$M_{Y}$ ; 3a) $A_{1}$→$M_{O2}$ and $O_{1}$→$M_{O2}$; and 4a) $O_{2}$→$M_{A2}$.

^d^ 200 bootstraps were used to obtain bootstrap model-based SEs and coverage.

**Table S4: Summary statistics for stage 1 treatment effect estimate for Treatment effect 4 simulation setting. (True value=-0.01) Note due to long computational time we have selected to present 3 treatment effect simulation settings and of those 2 missing data scenarios for the MI estimates.**

|  | Complete Case Analysis | | | | | Multiple Imputation | | | | |
| --- | --- | --- | --- | --- | --- | --- | --- | --- | --- | --- |
| Missing data scenarios | **Bias** | **Empirical S.E** | **Bootstrap Model-based S.E** | **MSE** | **Coverage** | **Bias** | **Empirical S.E** | **Bootstrap Model-based S.E** | **MSE** | **Coverage** |
|  | **m-DAG 1:** $\boldsymbol{O}_{\boldsymbol{2}}$ **and** $\boldsymbol{Y}$ **are missing not dependent on other variables** | | | | | | | | | |
| 20% missingness | -0.0398 | 0.0707 | 0.0738 | 0.0066 | 0.895 | -0.038 | 0.0635 | 0.066 | 0.0055 | 0.874 |
| 40% missingness | -0.0428 | 0.0958 | 0.0993 | 0.011 | 0.896 | ~~-~~ | - | - | - | - |
|  | **m-DAG 2a:** $\boldsymbol{O}_{\boldsymbol{2}}$**is missing not dependent on other variables and** $\boldsymbol{Y}$ **missing dependent on** $\boldsymbol{A}_{\boldsymbol{2}}$ | | | | | | | | | |
| 20% missingness + weak association | -0.0332 | 0.0634 | 0.0665 | 0.0051 | 0.894 | ~~-~~ | - | - | - | - |
| 20% missingness + strong association | -0.0326 | 0.0632 | 0.0665 | 0.0051 | 0.894 | ~~-~~ | - | - | - | - |
| 40% missingness + weak association | -0.0394 | 0.0751 | 0.0771 | 0.0072 | 0.888 | ~~-~~ | - | - | - | - |
| 40% missingness + strong association | -0.0364 | 0.0775 | 0.0775 | 0.0073 | 0.875 | ~~-~~ | - | - | - | - |
|  | **m-DAG 3a:** $\boldsymbol{O}_{\boldsymbol{2}}$ **missing dependent on** $\boldsymbol{A}_{\boldsymbol{1}}$ **and** $\boldsymbol{O}_{\boldsymbol{1}}$**;** $\boldsymbol{Y}$ **missing if** $\boldsymbol{O}_{\boldsymbol{2}}$ **missing.** | | | | | | | | | |
| 20% missingness + weak association | -0.0319 | 0.0642 | 0.0664 | 0.0051 | 0.892 | ~~-~~ | - | - | - | - |
| 20% missingness + strong association | -0.0288 | 0.0647 | 0.0670 | 0.0050 | 0.910 | ~~-~~ | - | - | - | - |
| 40% missingness + weak association | -0.0351 | 0.0711 | 0.0754 | 0.0063 | 0.908 | ~~-~~ | - | - | - | - |
| 40% missingness + strong association | -0.0315 | 0.0817 | 0.0827 | 0.0077 | 0.904 | ~~-~~ | - | - | - | - |
|  | **m-DAG 4a:** $\boldsymbol{A}_{\boldsymbol{2}}$ **missing dependent on** $\boldsymbol{O}_{\boldsymbol{2}}$ **and** $\boldsymbol{Y}$ **missing if** $\boldsymbol{A}_{\boldsymbol{2}}$**missing.** | | | | | | | | | |
| 20% missingness + weak association | -0.0313 | 0.0607 | 0.0641 | 0.0047 | 0.889 | ~~-~~ | - | - | - | - |
| 20% missingness + strong association | -0.0302 | 0.0625 | 0.0649 | 0.0048 | 0.887 | ~~-~~ | - | - | - | - |
| 40% missingness + weak association | -0.0374 | 0.0717 | 0.0746 | 0.0065 | 0.886 | ~~-~~ | - | - | - | - |
| 40% missingness + strong association | -0.0329 | 0.0699 | 0.0721 | 0.0060 | 0.894 | ~~-~~ | - | - | - | - |

Footnotes: ^a^ Treatment effect 4 simulation setting when there is a very weak treatment effect at stage 2 for half of the participants, but a relatively large effect for the other half.

^b^$O_{1}$= basic characteristic; $A_{1}$= stage 1 treatment; $O_{2}$= stage 1 responder status; $A_{2}$= stage 2 treatment; $Y$ = stage 2 outcome

^c^ For weak association between the missing indicator and other variables (as described below) an OR of 1.6 was used; and for a strong association an OR of 3 was used. The other variables were: missing data scenario 2a) $A_{2}$→$M_{Y}$ ; 3a) $A_{1}$→$M_{O2}$ and $O_{1}$→$M_{O2}$; and 4a) $O_{2}$→$M_{A2}$.

^d^ 200 bootstraps were used to obtain bootstrap model-based SEs and coverage.

**Table S5: Summary statistics for stage 2 treatment effect estimate for Treatment effect 1 simulation setting. (True value=0)**

|  | Complete Case Analysis | | | | | Multiple Imputation | | | | |
| --- | --- | --- | --- | --- | --- | --- | --- | --- | --- | --- |
| Missing data scenarios | **Bias** | **Empirical S.E** | **Model-based S.E** | **MSE** | **Coverage** | **Bias** | **Empirical S.E** | **Model-based S.E** | **MSE** | **Coverage** |
|  | **m-DAG 1:** $\boldsymbol{O}_{\boldsymbol{2}}$ **and** $\boldsymbol{Y}$ **are missing not dependent on other variables** | | | | | | | | | |
| 20% missingness | -0.0022 | 0.0555 | 0.0564 | 0.0031 | 0.946 | -0.0021 | 0.0487 | 0.0508 | 0.0024 | 0.959 |
| 40% missingness | -0.0019 | 0.077 | 0.0755 | 0.0059 | 0.938 | 0 | 0.0575 | 0.0587 | 0.0033 | 0.953 |
|  | **m-DAG 2a:** $\boldsymbol{O}_{\boldsymbol{2}}$**is missing not dependent on other variables and** $\boldsymbol{Y}$ **missing dependent on** $\boldsymbol{A}_{\boldsymbol{2}}$ | | | | | | | | | |
| 20% missingness + weak association | -0.0022 | 0.0504 | 0.0505 | 0.0025 | 0.953 | -0.0012 | 0.0502 | 0.0506 | 0.0025 | 0.956 |
| 20% missingness + strong association | -0.0006 | 0.0488 | 0.0506 | 0.0024 | 0.960 | -0.0011 | 0.0500 | 0.0506 | 0.0025 | 0.949 |
| 40% missingness + weak association | -0.0017 | 0.0573 | 0.0588 | 0.0033 | 0.955 | 0.0013 | 0.0588 | 0.0587 | 0.0035 | 0.959 |
| 40% missingness + strong association | -0.0019 | 0.0575 | 0.0590 | 0.0033 | 0.954 | -0.0017 | 0.0561 | 0.0591 | 0.0031 | 0.966 |
|  | **m-DAG 2b:** $\boldsymbol{O}_{\boldsymbol{2}}$ **missing dependent on a common cause between** $\boldsymbol{M}_{\boldsymbol{O}\boldsymbol{2}}$ **and** $\boldsymbol{Y}$ **which induces an association between** $\boldsymbol{M}_{\boldsymbol{O}\boldsymbol{2}}$**and** $\boldsymbol{Y}$**;** $\boldsymbol{Y}$ **missing dependent on** $\boldsymbol{A}_{\boldsymbol{2}}$ **and if** $\boldsymbol{O}_{\boldsymbol{2}}$ **is missing.** | | | | | | | | | |
| 20% missingness + weak association | -0.0012 | 0.0493 | 0.0501 | 0.0024 | 0.944 | -0.0014 | 0.0494 | 0.0501 | 0.0024 | 0.950 |
| 20% missingness + strong association | -0.0013 | 0.0469 | 0.0476 | 0.0022 | 0.955 | -0.0018 | 0.0476 | 0.0477 | 0.0023 | 0.949 |
| 40% missingness + weak association | -0.0007 | 0.0562 | 0.0588 | 0.0032 | 0.959 | -0.0021 | 0.0570 | 0.0589 | 0.0033 | 0.959 |
| 40% missingness + strong association | -0.0001 | 0.0519 | 0.0533 | 0.0027 | 0.955 | -0.0008 | 0.0517 | 0.0533 | 0.0027 | 0.955 |
|  | **m-DAG 3a:** $\boldsymbol{O}_{\boldsymbol{2}}$ **missing dependent on** $\boldsymbol{A}_{\boldsymbol{1}}$ **and** $\boldsymbol{O}_{\boldsymbol{1}}$**;** $\boldsymbol{Y}$ **missing if** $\boldsymbol{O}_{\boldsymbol{2}}$ **missing.** | | | | | | | | | |
| 20% missingness + weak association | -0.0018 | 0.0490 | 0.0501 | 0.0024 | 0.954 | -0.0005 | 0.0489 | 0.0501 | 0.0024 | 0.958 |
| 20% missingness + strong association | -0.0006 | 0.0492 | 0.0499 | 0.0024 | 0.962 | -0.0010 | 0.0487 | 0.0499 | 0.0024 | 0.955 |
| 40% missingness + weak association | -0.0009 | 0.0566 | 0.0565 | 0.0032 | 0.945 | -0.0014 | 0.0547 | 0.0565 | 0.0030 | 0.951 |
| 40% missingness + strong association | -0.0015 | 0.0568 | 0.0578 | 0.0032 | 0.949 | -0.0002 | 0.0580 | 0.0579 | 0.0034 | 0.949 |
|  | **m-DAG 3b:** $\boldsymbol{O}_{\boldsymbol{2}}$ **missing dependent on** $\boldsymbol{A}_{\boldsymbol{1}}$**,** $\boldsymbol{O}_{\boldsymbol{1}}$**and a common cause between** $\boldsymbol{M}_{\boldsymbol{O}\boldsymbol{2}}$**and** $\boldsymbol{Y}$ **which induces an association between** $\boldsymbol{M}_{\boldsymbol{O}\boldsymbol{2}}$**and** $\boldsymbol{Y}$**;** $\boldsymbol{Y}$ **missing if** $\boldsymbol{O}_{\boldsymbol{2}}$ **missing.** | | | | | | | | | |
| 20% missingness + weak association | -0.0004 | 0.0478 | 0.0497 | 0.0023 | 0.950 | -0.0013 | 0.0485 | 0.0496 | 0.0024 | 0.964 |
| 20% missingness + strong association | -0.0012 | 0.0480 | 0.0493 | 0.0023 | 0.959 | -0.0012 | 0.0477 | 0.0494 | 0.0023 | 0.964 |
| 40% missingness + weak association | -0.0002 | 0.0546 | 0.0565 | 0.0030 | 0.951 | 0.0005 | 0.0564 | 0.0565 | 0.0032 | 0.953 |
| 40% missingness + strong association | -0.0011 | 0.0546 | 0.0546 | 0.0030 | 0.945 | 0.0002 | 0.0535 | 0.0546 | 0.0029 | 0.948 |
|  | **m-DAG 4a:** $\boldsymbol{A}_{\boldsymbol{2}}$ **missing dependent on** $\boldsymbol{O}_{\boldsymbol{2}}$ **and** $\boldsymbol{Y}$ **missing if** $\boldsymbol{A}_{\boldsymbol{2}}$**missing.** | | | | | | | | | |
| 20% missingness + weak association | -0.0020 | 0.0473 | 0.0490 | 0.0022 | 0.952 | 0.0001 | 0.0467 | 0.0490 | 0.0022 | 0.958 |
| 20% missingness + strong association | -0.0015 | 0.0487 | 0.0498 | 0.0024 | 0.962 | -0.0003 | 0.0490 | 0.0499 | 0.0024 | 0.951 |
| 40% missingness + weak association | -0.0024 | 0.0551 | 0.0572 | 0.0030 | 0.952 | -0.0021 | 0.0554 | 0.0573 | 0.0031 | 0.957 |
| 40% missingness + strong association | 0.0013 | 0.0573 | 0.0573 | 0.0033 | 0.951 | -0.0009 | 0.0564 | 0.0573 | 0.0032 | 0.954 |
|  | **m-DAG 4b:** $\boldsymbol{A}_{\boldsymbol{2}}$ **missing dependent on** $\boldsymbol{O}_{\boldsymbol{2}}$ **and** $\boldsymbol{Y}$ **(assuming a common cause of missingness in** $\boldsymbol{A}_{\boldsymbol{2}}$ **and** $\boldsymbol{Y}$**);** $\boldsymbol{Y}$ **missing if** $\boldsymbol{A}_{\boldsymbol{2}}$ **missing.** | | | | | | | | | |
| 20% missingness + weak association | -0.0024 | 0.0478 | 0.0489 | 0.0023 | 0.952 | -0.0008 | 0.0476 | 0.0490 | 0.0023 | 0.954 |
| 20% missingness + strong association | -0.0006 | 0.0458 | 0.0472 | 0.0021 | 0.953 | -0.0019 | 0.0463 | 0.0472 | 0.0021 | 0.953 |
| 40% missingness + weak association | -0.0016 | 0.0525 | 0.0544 | 0.0028 | 0.958 | -0.0009 | 0.0533 | 0.0544 | 0.0028 | 0.959 |
| 40% missingness + strong association | -0.0002 | 0.0500 | 0.0532 | 0.0025 | 0.964 | -0.0016 | 0.0511 | 0.0533 | 0.0026 | 0.963 |

Footnotes: ^a^ Treatment effect 1 simulation setting when there is no treatment effect at either stage 1 or 2 for any participant.

^b^ $O_{1}$= basic characteristic; $A_{1}$= stage 1 treatment; $O_{2}$= stage 1 responder status; $A_{2}$= stage 2 treatment; $Y$ = stage 2 outcome

^c^ For weak association between the missing indicator and other variables (as described below) an OR of 1.6 was used; and for a strong association an OR of 3 was used. The other variables were: missing data scenario 2a) $A_{2}$→$M_{Y}$ ; 2b) $Y$ →$O_{2}$ and $A_{2}$→$M_{Y}$; 3a) $A_{1}$→$M_{O2}$ and $O_{1}$→$M_{O2}$; 3b) $A_{1}$→$M_{O2}$ , $O_{1}$→$M_{O2}$ and $Y$ →$M_{O2}$; 4a) $O_{2}$→$M_{A2}$; and 4b) $O_{2}$→$M_{A2}$ and $Y$ →$M_{A2}$.

**Table S6: Summary statistics for stage 2 treatment effect estimate for Treatment effect 2 simulation setting . (True value=0)**

|  | Complete Case Analysis | | | | | Multiple Imputation | | | | |
| --- | --- | --- | --- | --- | --- | --- | --- | --- | --- | --- |
| Missing data scenarios | **Bias** | **Empirical S.E** | **Model-based S.E** | **MSE** | **Coverage** | **Bias** | **Empirical S.E** | **Model-based S.E** | **MSE** | **Coverage** |
|  | **m-DAG 1:** $\boldsymbol{O}_{\boldsymbol{2}}$ **and** $\boldsymbol{Y}$ **are missing not dependent on other variables** | | | | | | | | | |
| 20% missingness | 0.0001 | 0.0551 | 0.0565 | 0.0030 | 0.956 | 0.0002 | 0.0508 | 0.0507 | 0.0026 | 0.944 |
| 40% missingness | -0.0037 | 0.0782 | 0.0759 | 0.0061 | 0.942 | -0.0001 | 0.0609 | 0.0589 | 0.0037 | 0.942 |
|  | **m-DAG 2a:** $\boldsymbol{O}_{\boldsymbol{2}}$**is missing not dependent on other variables and** $\boldsymbol{Y}$ **missing dependent on** $\boldsymbol{A}_{\boldsymbol{2}}$ | | | | | | | | | |
| 20% missingness + weak association | -0.0024 | 0.0514 | 0.0506 | 0.0026 | 0.941 | -0.0009 | 0.0503 | 0.0505 | 0.0025 | 0.955 |
| 20% missingness + strong association | -0.0014 | 0.0516 | 0.0507 | 0.0027 | 0.945 | -0.0011 | 0.0515 | 0.0507 | 0.0027 | 0.942 |
| 40% missingness + weak association | -0.0011 | 0.0599 | 0.0588 | 0.0036 | 0.944 | -0.0002 | 0.0575 | 0.0588 | 0.0033 | 0.959 |
| 40% missingness + strong association | -0.0009 | 0.0590 | 0.0591 | 0.0035 | 0.957 | -0.0017 | 0.0592 | 0.0591 | 0.0035 | 0.950 |
|  | **m-DAG 2b:** $\boldsymbol{O}_{\boldsymbol{2}}$ **missing dependent on a common cause between** $\boldsymbol{M}_{\boldsymbol{O}\boldsymbol{2}}$ **and** $\boldsymbol{Y}$ **which induces an association between** $\boldsymbol{M}_{\boldsymbol{O}\boldsymbol{2}}$**and** $\boldsymbol{Y}$**;** $\boldsymbol{Y}$ **missing dependent on** $\boldsymbol{A}_{\boldsymbol{2}}$ **and if** $\boldsymbol{O}_{\boldsymbol{2}}$ **is missing.** | | | | | | | | | |
| 20% missingness + weak association | 0.0037 | 0.0515 | 0.0508 | 0.0027 | 0.944 | 0.0032 | 0.0508 | 0.0509 | 0.0026 | 0.956 |
| 20% missingness + strong association | 0.0283 | 0.0509 | 0.0508 | 0.0034 | 0.924 | 0.0257 | 0.0507 | 0.0509 | 0.0032 | 0.932 |
| 40% missingness + weak association | 0.0012 | 0.0603 | 0.0605 | 0.0036 | 0.947 | 0.0033 | 0.0597 | 0.0606 | 0.0036 | 0.958 |
| 40% missingness + strong association | 0.0139 | 0.0587 | 0.0603 | 0.0036 | 0.952 | 0.0167 | 0.0600 | 0.0604 | 0.0039 | 0.945 |
|  | **m-DAG 3a:** $\boldsymbol{O}_{\boldsymbol{2}}$ **missing dependent on** $\boldsymbol{A}_{\boldsymbol{1}}$ **and** $\boldsymbol{O}_{\boldsymbol{1}}$**;** $\boldsymbol{Y}$ **missing if** $\boldsymbol{O}_{\boldsymbol{2}}$ **missing.** | | | | | | | | | |
| 20% missingness + weak association | -0.0001 | 0.0499 | 0.0502 | 0.0025 | 0.948 | -0.0001 | 0.0505 | 0.0502 | 0.0025 | 0.952 |
| 20% missingness + strong association | -0.0015 | 0.0494 | 0.0499 | 0.0024 | 0.953 | -0.0007 | 0.0507 | 0.0499 | 0.0026 | 0.948 |
| 40% missingness + weak association | -0.0009 | 0.0595 | 0.0566 | 0.0035 | 0.944 | -0.0001 | 0.0551 | 0.0567 | 0.0030 | 0.957 |
| 40% missingness + strong association | 0.0002 | 0.0552 | 0.0580 | 0.0031 | 0.970 | 0.0009 | 0.0592 | 0.0579 | 0.0035 | 0.945 |
|  | **m-DAG 3b:** $\boldsymbol{O}_{\boldsymbol{2}}$ **missing dependent on** $\boldsymbol{A}_{\boldsymbol{1}}$**,** $\boldsymbol{O}_{\boldsymbol{1}}$**and a common cause between** $\boldsymbol{M}_{\boldsymbol{O}\boldsymbol{2}}$**and** $\boldsymbol{Y}$ **which induces an association between** $\boldsymbol{M}_{\boldsymbol{O}\boldsymbol{2}}$**and** $\boldsymbol{Y}$**;** $\boldsymbol{Y}$ **missing if** $\boldsymbol{O}_{\boldsymbol{2}}$ **missing.** | | | | | | | | | |
| 20% missingness + weak association | -0.0011 | 0.0502 | 0.0497 | 0.0025 | 0.944 | -0.0007 | 0.0489 | 0.0496 | 0.0024 | 0.957 |
| 20% missingness + strong association | -0.0005 | 0.0494 | 0.0493 | 0.0024 | 0.949 | -0.0002 | 0.0491 | 0.0494 | 0.0024 | 0.951 |
| 40% missingness + weak association | 0.0013 | 0.0551 | 0.0566 | 0.0030 | 0.950 | -0.0020 | 0.0570 | 0.0566 | 0.0033 | 0.949 |
| 40% missingness + strong association | -0.0011 | 0.0546 | 0.0547 | 0.0030 | 0.954 | -0.0022 | 0.0544 | 0.0548 | 0.0030 | 0.952 |
|  | **m-DAG 4a:** $\boldsymbol{A}_{\boldsymbol{2}}$ **missing dependent on** $\boldsymbol{O}_{\boldsymbol{2}}$ **and** $\boldsymbol{Y}$ **missing if** $\boldsymbol{A}_{\boldsymbol{2}}$**missing.** | | | | | | | | | |
| 20% missingness + weak association | -0.0006 | 0.0500 | 0.0490 | 0.0025 | 0.950 | -0.0015 | 0.0497 | 0.0491 | 0.0025 | 0.947 |
| 20% missingness + strong association | -0.0012 | 0.0502 | 0.0499 | 0.0025 | 0.944 | -0.0007 | 0.0507 | 0.0499 | 0.0026 | 0.945 |
| 40% missingness + weak association | -0.0011 | 0.0591 | 0.0574 | 0.0035 | 0.945 | -0.0002 | 0.0582 | 0.0573 | 0.0034 | 0.949 |
| 40% missingness + strong association | 0.0004 | 0.0564 | 0.0574 | 0.0032 | 0.952 | 0.0012 | 0.0578 | 0.0573 | 0.0033 | 0.948 |
|  | **m-DAG 4b:** $\boldsymbol{A}_{\boldsymbol{2}}$ **missing dependent on** $\boldsymbol{O}_{\boldsymbol{2}}$ **and** $\boldsymbol{Y}$ **(assuming a common cause of missingness in** $\boldsymbol{A}_{\boldsymbol{2}}$ **and** $\boldsymbol{Y}$**);** $\boldsymbol{Y}$ **missing if** $\boldsymbol{A}_{\boldsymbol{2}}$ **missing.** | | | | | | | | | |
| 20% missingness + weak association | 0.0013 | 0.0490 | 0.0495 | 0.0024 | 0.960 | 0.0008 | 0.0502 | 0.0495 | 0.0025 | 0.949 |
| 20% missingness + strong association | 0.0030 | 0.0490 | 0.0488 | 0.0024 | 0.951 | 0.0028 | 0.0477 | 0.0488 | 0.0023 | 0.948 |
| 40% missingness + weak association | 0.0008 | 0.0559 | 0.0554 | 0.0031 | 0.946 | 0.0012 | 0.055 | 0.0553 | 0.003 | 0.951 |
| 40% missingness + strong association | -0.0002 | 0.0553 | 0.0562 | 0.0031 | 0.951 | -0.0003 | 0.0568 | 0.0562 | 0.0032 | 0.944 |

Footnotes: ^a^ Treatment effect 2 simulation setting when there is a relatively large treatment effect at stage 2 for every participant.

^b^ $O_{1}$= basic characteristic; $A_{1}$= stage 1 treatment; $O_{2}$= stage 1 responder status; $A_{2}$= stage 2 treatment; $Y$ = stage 2 outcome

^c^ For weak association between the missing indicator and other variables (as described below) an OR of 1.6 was used; and for a strong association an OR of 3 was used. The other variables were: missing data scenario 2a) $A_{2}$→$M_{Y}$ ; 2b) $Y$ →$O_{2}$ and $A_{2}$→$M_{Y}$; 3a) $A_{1}$→$M_{O2}$ and $O_{1}$→$M_{O2}$; 3b) $A_{1}$→$M_{O2}$ , $O_{1}$→$M_{O2}$ and $Y$ →$M_{O2}$; 4a) $O_{2}$→$M_{A2}$; and 4b) $O_{2}$→$M_{A2}$ and $Y$ →$M_{A2}$.

**Table S7: Summary statistics for stage 3 treatment effect estimate for Treatment effect 3 simulation setting. (True value=0.5)**

|  | Complete Case Analysis | | | | | Multiple Imputation | | | | |
| --- | --- | --- | --- | --- | --- | --- | --- | --- | --- | --- |
| Missing data scenarios | **Bias** | **Empirical S.E** | **Model-based S.E** | **MSE** | **Coverage** | **Bias** | **Empirical S.E** | **Model-based S.E** | **MSE** | **Coverage** |
|  | **m-DAG 1:** $\boldsymbol{O}_{\boldsymbol{2}}$ **and** $\boldsymbol{Y}$ **are missing not dependent on other variables** | | | | | | | | | |
| 20% missingness | 0.0012 | 0.0588 | 0.0563 | 0.0035 | 0.936 | 0.0008 | 0.0527 | 0.0507 | 0.0028 | 0.940 |
| 40% missingness | 0.0005 | 0.0759 | 0.0759 | 0.0058 | 0.948 | -0.0013 | 0.059 | 0.0589 | 0.0035 | 0.950 |
|  | **m-DAG 2a:** $\boldsymbol{O}_{\boldsymbol{2}}$**is missing not dependent on other variables and** $\boldsymbol{Y}$ **missing dependent on** $\boldsymbol{A}_{\boldsymbol{2}}$ | | | | | | | | | |
| 20% missingness + weak association | -0.0008 | 0.0528 | 0.0505 | 0.0028 | 0.943 | -0.0012 | 0.0528 | 0.0506 | 0.0028 | 0.939 |
| 20% missingness + strong association | -0.0006 | 0.0528 | 0.0506 | 0.0028 | 0.938 | 0.0008 | 0.0518 | 0.0506 | 0.0027 | 0.941 |
| 40% missingness + weak association | -0.0013 | 0.0593 | 0.0588 | 0.0035 | 0.952 | 0 | 0.0598 | 0.0588 | 0.0036 | 0.948 |
| 40% missingness + strong association | 0.0002 | 0.0598 | 0.0590 | 0.0036 | 0.947 | 0.0011 | 0.0607 | 0.0591 | 0.0037 | 0.944 |
|  | **m-DAG 2b:** $\boldsymbol{O}_{\boldsymbol{2}}$ **missing dependent on a common cause between** $\boldsymbol{M}_{\boldsymbol{O}\boldsymbol{2}}$ **and** $\boldsymbol{Y}$ **which induces an association between** $\boldsymbol{M}_{\boldsymbol{O}\boldsymbol{2}}$**and** $\boldsymbol{Y}$**;** $\boldsymbol{Y}$ **missing dependent on** $\boldsymbol{A}_{\boldsymbol{2}}$ **and if** $\boldsymbol{O}_{\boldsymbol{2}}$ **is missing.** | | | | | | | | | |
| 20% missingness + weak association | -0.0152 | 0.0533 | 0.0504 | 0.0031 | 0.928 | -0.0138 | 0.0531 | 0.0504 | 0.0030 | 0.933 |
| 20% missingness + strong association | -0.0508 | 0.0508 | 0.0489 | 0.0052 | 0.814 | -0.0504 | 0.0498 | 0.0489 | 0.0050 | 0.825 |
| 40% missingness + weak association | -0.0207 | 0.0620 | 0.0597 | 0.0043 | 0.923 | -0.0212 | 0.0599 | 0.0598 | 0.004 | 0.929 |
| 40% missingness + strong association | -0.0789 | 0.0566 | 0.0565 | 0.0094 | 0.703 | -0.0753 | 0.0561 | 0.0565 | 0.0088 | 0.737 |
|  | **m-DAG 3a:** $\boldsymbol{O}_{\boldsymbol{2}}$ **missing dependent on** $\boldsymbol{A}_{\boldsymbol{1}}$ **and** $\boldsymbol{O}_{\boldsymbol{1}}$**;** $\boldsymbol{Y}$ **missing if** $\boldsymbol{O}_{\boldsymbol{2}}$ **missing.** | | | | | | | | | |
| 20% missingness + weak association | 0.0003 | 0.0523 | 0.0502 | 0.0027 | 0.945 | 0.0003 | 0.0521 | 0.0501 | 0.0027 | 0.939 |
| 20% missingness + strong association | -0.0004 | 0.0507 | 0.0498 | 0.0026 | 0.949 | -0.0007 | 0.0515 | 0.0499 | 0.0026 | 0.951 |
| 40% missingness + weak association | 0.0021 | 0.0590 | 0.0565 | 0.0035 | 0.940 | -0.0001 | 0.0588 | 0.0566 | 0.0035 | 0.946 |
| 40% missingness + strong association | 0.0006 | 0.0600 | 0.0580 | 0.0036 | 0.937 | 0.0011 | 0.0598 | 0.0579 | 0.0036 | 0.948 |
|  | **m-DAG 3b:** $\boldsymbol{O}_{\boldsymbol{2}}$ **missing dependent on** $\boldsymbol{A}_{\boldsymbol{1}}$**,** $\boldsymbol{O}_{\boldsymbol{1}}$**and a common cause between** $\boldsymbol{M}_{\boldsymbol{O}\boldsymbol{2}}$**and** $\boldsymbol{Y}$ **which induces an association between** $\boldsymbol{M}_{\boldsymbol{O}\boldsymbol{2}}$**and** $\boldsymbol{Y}$**;** $\boldsymbol{Y}$ **missing if** $\boldsymbol{O}_{\boldsymbol{2}}$ **missing.** | | | | | | | | | |
| 20% missingness + weak association | -0.0015 | 0.0521 | 0.0497 | 0.0027 | 0.942 | -0.0003 | 0.0513 | 0.0497 | 0.0026 | 0.947 |
| 20% missingness + strong association | 0.0005 | 0.0522 | 0.0493 | 0.0027 | 0.940 | 0.0001 | 0.0505 | 0.0494 | 0.0026 | 0.941 |
| 40% missingness + weak association | 0.0009 | 0.0602 | 0.0565 | 0.0036 | 0.934 | 0.0003 | 0.0593 | 0.0565 | 0.0035 | 0.938 |
| 40% missingness + strong association | -0.0004 | 0.0558 | 0.0548 | 0.0031 | 0.945 | 0.0004 | 0.0564 | 0.0548 | 0.0032 | 0.939 |
|  | **m-DAG 4a:** $\boldsymbol{A}_{\boldsymbol{2}}$ **missing dependent on** $\boldsymbol{O}_{\boldsymbol{2}}$ **and** $\boldsymbol{Y}$ **missing if** $\boldsymbol{A}_{\boldsymbol{2}}$**missing.** | | | | | | | | | |
| 20% missingness + weak association | -0.0011 | 0.0522 | 0.0490 | 0.0027 | 0.938 | 0.0007 | 0.0516 | 0.0490 | 0.0027 | 0.940 |
| 20% missingness + strong association | -0.0007 | 0.0522 | 0.0499 | 0.0027 | 0.943 | -0.0006 | 0.0530 | 0.0498 | 0.0028 | 0.932 |
| 40% missingness + weak association | -0.0014 | 0.0604 | 0.0573 | 0.0036 | 0.933 | -0.0021 | 0.0598 | 0.0573 | 0.0036 | 0.948 |
| 40% missingness + strong association | -0.0004 | 0.0610 | 0.0574 | 0.0037 | 0.937 | 0.0004 | 0.0596 | 0.0573 | 0.0036 | 0.938 |
|  | **m-DAG 4b:** $\boldsymbol{A}_{\boldsymbol{2}}$ **missing dependent on** $\boldsymbol{O}_{\boldsymbol{2}}$ **and** $\boldsymbol{Y}$ **(assuming a common cause of missingness in** $\boldsymbol{A}_{\boldsymbol{2}}$ **and** $\boldsymbol{Y}$**);** $\boldsymbol{Y}$ **missing if** $\boldsymbol{A}_{\boldsymbol{2}}$ **missing.** | | | | | | | | | |
| 20% missingness + weak association | -0.0132 | 0.0519 | 0.0492 | 0.0029 | 0.932 | -0.0127 | 0.0514 | 0.0492 | 0.0028 | 0.933 |
| 20% missingness + strong association | -0.0411 | 0.0506 | 0.0480 | 0.0042 | 0.839 | -0.0401 | 0.0499 | 0.0479 | 0.0041 | 0.857 |
| 40% missingness + weak association | -0.0191 | 0.0565 | 0.0548 | 0.0036 | 0.929 | -0.0182 | 0.0554 | 0.0548 | 0.0034 | 0.943 |
| 40% missingness + strong association | -0.0598 | 0.0553 | 0.0544 | 0.0066 | 0.802 | -0.0577 | 0.0552 | 0.0545 | 0.0064 | 0.823 |

Footnotes: ^a^ Treatment effect 3 simulation setting when there is no treatment effect at stage 2 for half of the participants, but a relatively large effect for the other half.

^b^ $O_{1}$= basic characteristic; $A_{1}$= stage 1 treatment; $O_{2}$= stage 1 responder status; $A_{2}$= stage 2 treatment; $Y$ = stage 2 outcome

^c^ For weak association between the missing indicator and other variables (as described below) an OR of 1.6 was used; and for a strong association an OR of 3 was used. The other variables were: missing data scenario 2a) $A_{2}$→$M_{Y}$ ; 2b) $Y$ →$O_{2}$ and $A_{2}$→$M_{Y}$; 3a) $A_{1}$→$M_{O2}$ and $O_{1}$→$M_{O2}$; 3b) $A_{1}$→$M_{O2}$ , $O_{1}$→$M_{O2}$ and $Y$ →$M_{O2}$; 4a) $O_{2}$→$M_{A2}$; and 4b) $O_{2}$→$M_{A2}$ and $Y$ →$M_{A2}$.

**Table S8: Summary statistics for stage 2 treatment effect estimate for Treatment effect 4 simulation setting. (True value=0.5)**

|  | Complete Case Analysis | | | | | Multiple Imputation | | | | |
| --- | --- | --- | --- | --- | --- | --- | --- | --- | --- | --- |
| Missing data scenarios | **Bias** | **Empirical S.E** | **Model-based S.E** | **MSE** | **Coverage** | **Bias** | **Empirical S.E** | **Model-based S.E** | **MSE** | **Coverage** |
|  | **m-DAG 1:** $\boldsymbol{O}_{\boldsymbol{2}}$ **and** $\boldsymbol{Y}$ **are missing not dependent on other variables** | | | | | | | | | |
| 20% missingness | -0.0032 | 0.0549 | 0.0563 | 0.0030 | 0.957 | -0.003 | 0.0499 | 0.0505 | 0.0025 | 0.954 |
| 40% missingness | -0.0028 | 0.0758 | 0.0756 | 0.0058 | 0.960 | -0.0026 | 0.0576 | 0.0588 | 0.0033 | 0.951 |
|  | **m-DAG 2a:** $\boldsymbol{O}_{\boldsymbol{2}}$**is missing not dependent on other variables and** $\boldsymbol{Y}$ **missing dependent on** $\boldsymbol{A}_{\boldsymbol{2}}$ | | | | | | | | | |
| 20% missingness + weak association | 0.0001 | 0.0511 | 0.0505 | 0.0026 | 0.946 | -0.0005 | 0.0499 | 0.0505 | 0.0025 | 0.961 |
| 20% missingness + strong association | -0.0022 | 0.0501 | 0.0507 | 0.0025 | 0.957 | -0.0008 | 0.0496 | 0.0506 | 0.0025 | 0.952 |
| 40% missingness + weak association | -0.0014 | 0.0577 | 0.0587 | 0.0033 | 0.947 | -0.0019 | 0.0594 | 0.0588 | 0.0035 | 0.945 |
| 40% missingness + strong association | -0.0030 | 0.0575 | 0.0591 | 0.0033 | 0.956 | -0.0019 | 0.0577 | 0.0591 | 0.0033 | 0.957 |
|  | **m-DAG 2b:** $\boldsymbol{O}_{\boldsymbol{2}}$ **missing dependent on a common cause between** $\boldsymbol{M}_{\boldsymbol{O}\boldsymbol{2}}$ **and** $\boldsymbol{Y}$ **which induces an association between** $\boldsymbol{M}_{\boldsymbol{O}\boldsymbol{2}}$**and** $\boldsymbol{Y}$**;** $\boldsymbol{Y}$ **missing dependent on** $\boldsymbol{A}_{\boldsymbol{2}}$ **and if** $\boldsymbol{O}_{\boldsymbol{2}}$ **is missing.** | | | | | | | | | |
| 20% missingness + weak association | -0.0170 | 0.0502 | 0.0504 | 0.0028 | 0.943 | -0.0179 | 0.0502 | 0.0504 | 0.0028 | 0.939 |
| 20% missingness + strong association | -0.0538 | 0.0498 | 0.0490 | 0.0054 | 0.802 | -0.0523 | 0.0483 | 0.0490 | 0.0051 | 0.817 |
| 40% missingness + weak association | -0.0253 | 0.0602 | 0.0597 | 0.0043 | 0.929 | -0.0261 | 0.0583 | 0.0598 | 0.0041 | 0.931 |
| 40% missingness + strong association | -0.0804 | 0.0543 | 0.0565 | 0.0094 | 0.702 | -0.0817 | 0.0560 | 0.0564 | 0.0098 | 0.694 |
|  | **m-DAG 3a:** $\boldsymbol{O}_{\boldsymbol{2}}$ **missing dependent on** $\boldsymbol{A}_{\boldsymbol{1}}$ **and** $\boldsymbol{O}_{\boldsymbol{1}}$**;** $\boldsymbol{Y}$ **missing if** $\boldsymbol{O}_{\boldsymbol{2}}$ **missing.** | | | | | | | | | |
| 20% missingness + weak association | -0.0009 | 0.0496 | 0.0501 | 0.0025 | 0.954 | -0.0017 | 0.0515 | 0.0501 | 0.0027 | 0.942 |
| 20% missingness + strong association | -0.0016 | 0.0494 | 0.0498 | 0.0024 | 0.955 | -0.0021 | 0.0502 | 0.0499 | 0.0025 | 0.944 |
| 40% missingness + weak association | -0.0025 | 0.0573 | 0.0565 | 0.0033 | 0.948 | -0.0015 | 0.0536 | 0.0565 | 0.0029 | 0.961 |
| 40% missingness + strong association | -0.0034 | 0.0583 | 0.0578 | 0.0034 | 0.948 | -0.0026 | 0.0592 | 0.0578 | 0.0035 | 0.944 |
|  | **m-DAG 3b:** $\boldsymbol{O}_{\boldsymbol{2}}$ **missing dependent on** $\boldsymbol{A}_{\boldsymbol{1}}$**,** $\boldsymbol{O}_{\boldsymbol{1}}$**and a common cause between** $\boldsymbol{M}_{\boldsymbol{O}\boldsymbol{2}}$**and** $\boldsymbol{Y}$ **which induces an association between** $\boldsymbol{M}_{\boldsymbol{O}\boldsymbol{2}}$**and** $\boldsymbol{Y}$**;** $\boldsymbol{Y}$ **missing if** $\boldsymbol{O}_{\boldsymbol{2}}$ **missing.** | | | | | | | | | |
| 20% missingness + weak association | -0.0015 | 0.0514 | 0.0496 | 0.0026 | 0.948 | -0.0018 | 0.0491 | 0.0496 | 0.0024 | 0.954 |
| 20% missingness + strong association | -0.0019 | 0.0499 | 0.0493 | 0.0025 | 0.945 | -0.0020 | 0.0491 | 0.0493 | 0.0024 | 0.955 |
| 40% missingness + weak association | -0.0014 | 0.0568 | 0.0565 | 0.0032 | 0.946 | -0.0012 | 0.0576 | 0.0565 | 0.0033 | 0.948 |
| 40% missingness + strong association | -0.0032 | 0.0559 | 0.0547 | 0.0031 | 0.946 | -0.0038 | 0.0563 | 0.0547 | 0.0032 | 0.947 |
|  | **m-DAG 4a:** $\boldsymbol{A}_{\boldsymbol{2}}$ **missing dependent on** $\boldsymbol{O}_{\boldsymbol{2}}$ **and** $\boldsymbol{Y}$ **missing if** $\boldsymbol{A}_{\boldsymbol{2}}$**missing.** | | | | | | | | | |
| 20% missingness + weak association | -0.0008 | 0.0492 | 0.0490 | 0.0024 | 0.954 | -0.0004 | 0.0494 | 0.0489 | 0.0024 | 0.951 |
| 20% missingness + strong association | -0.0019 | 0.0489 | 0.0498 | 0.0024 | 0.952 | -0.0013 | 0.0499 | 0.0498 | 0.0025 | 0.953 |
| 40% missingness + weak association | -0.0018 | 0.0559 | 0.0573 | 0.0031 | 0.958 | -0.0017 | 0.0585 | 0.0572 | 0.0034 | 0.947 |
| 40% missingness + strong association | -0.0005 | 0.0560 | 0.0574 | 0.0031 | 0.957 | 0.0002 | 0.0558 | 0.0573 | 0.0031 | 0.958 |
|  | **m-DAG 4b:** $\boldsymbol{A}_{\boldsymbol{2}}$ **missing dependent on** $\boldsymbol{O}_{\boldsymbol{2}}$ **and** $\boldsymbol{Y}$ **(assuming a common cause of missingness in** $\boldsymbol{A}_{\boldsymbol{2}}$ **and** $\boldsymbol{Y}$**);** $\boldsymbol{Y}$ **missing if** $\boldsymbol{A}_{\boldsymbol{2}}$ **missing.** | | | | | | | | | |
| 20% missingness + weak association | -0.0146 | 0.0488 | 0.0492 | 0.0026 | 0.948 | -0.0149 | 0.0487 | 0.0491 | 0.0026 | 0.944 |
| 20% missingness + strong association | -0.0432 | 0.0464 | 0.0479 | 0.0040 | 0.865 | -0.0418 | 0.0464 | 0.0479 | 0.0039 | 0.871 |
| 40% missingness + weak association | -0.0218 | 0.0559 | 0.0547 | 0.0036 | 0.922 | -0.0194 | 0.0534 | 0.0548 | 0.0032 | 0.944 |
| 40% missingness + strong association | -0.0594 | 0.0534 | 0.0546 | 0.0064 | 0.817 | -0.0608 | 0.0536 | 0.0545 | 0.0066 | 0.797 |

Footnotes: ^a^ Treatment effect 4 simulation setting when there is a very weak treatment effect at stage 2 for half of the participants, but a relatively large effect for the other half.

^b^ $O_{1}$= basic characteristic; $A_{1}$= stage 1 treatment; $O_{2}$= stage 1 responder status; $A_{2}$= stage 2 treatment; $Y$ = stage 2 outcome

^c^ For weak association between the missing indicator and other variables (as described below) an OR of 1.6 was used; and for a strong association an OR of 3 was used. The other variables were: missing data scenario 2a) $A_{2}$→$M_{Y}$ ; 2b) $Y$ →$O_{2}$ and $A_{2}$→$M_{Y}$; 3a) $A_{1}$→$M_{O2}$ and $O_{1}$→$M_{O2}$; 3b) $A_{1}$→$M_{O2}$ , $O_{1}$→$M_{O2}$ and $Y$ →$M_{O2}$; 4a) $O_{2}$→$M_{A2}$; and 4b) $O_{2}$→$M_{A2}$ and $Y$ →$M_{A2}$.

**Table S9: Summary statistics for stage 2 treatment effect estimate for Treatment effect 5 simulation setting. (True value=1)**

|  | Complete Case Analysis | | | | | Multiple Imputation | | | | |
| --- | --- | --- | --- | --- | --- | --- | --- | --- | --- | --- |
| Missing data scenarios | **Bias** | **Empirical S.E** | **Model-based S.E** | **MSE** | **Coverage** | **Bias** | **Empirical S.E** | **Model-based S.E** | **MSE** | **Coverage** |
|  | **m-DAG 1:** $\boldsymbol{O}_{\boldsymbol{2}}$ **and** $\boldsymbol{Y}$ **are missing not dependent on other variables** | | | | | | | | | |
| 20% missingness | 0.0001 | 0.0551 | 0.0565 | 0.0030 | 0.956 | 0.003 | 0.0487 | 0.0506 | 0.0024 | 0.959 |
| 40% missingness | -0.0037 | 0.0782 | 0.0759 | 0.0061 | 0.942 | 0.0035 | 0.0596 | 0.0589 | 0.0036 | 0.934 |
|  | **m-DAG 2a:** $\boldsymbol{O}_{\boldsymbol{2}}$**is missing not dependent on other variables and** $\boldsymbol{Y}$ **missing dependent on** $\boldsymbol{A}_{\boldsymbol{2}}$ | | | | | | | | | |
| 20% missingness + weak association | -0.0024 | 0.0514 | 0.0506 | 0.0026 | 0.941 | 0.0030 | 0.0500 | 0.0505 | 0.0025 | 0.942 |
| 20% missingness + strong association | -0.0014 | 0.0516 | 0.0507 | 0.0027 | 0.945 | 0.0029 | 0.0500 | 0.0506 | 0.0025 | 0.956 |
| 40% missingness + weak association | -0.0011 | 0.0599 | 0.0588 | 0.0036 | 0.944 | 0.0036 | 0.0570 | 0.0587 | 0.0033 | 0.95 |
| 40% missingness + strong association | -0.0009 | 0.0590 | 0.0591 | 0.0035 | 0.957 | 0.0025 | 0.0592 | 0.0591 | 0.0035 | 0.951 |
|  | **m-DAG 2b:** $\boldsymbol{O}_{\boldsymbol{2}}$ **missing dependent on a common cause between** $\boldsymbol{M}_{\boldsymbol{O}\boldsymbol{2}}$ **and** $\boldsymbol{Y}$ **which induces an association between** $\boldsymbol{M}_{\boldsymbol{O}\boldsymbol{2}}$**and** $\boldsymbol{Y}$**;** $\boldsymbol{Y}$ **missing dependent on** $\boldsymbol{A}_{\boldsymbol{2}}$ **and if** $\boldsymbol{O}_{\boldsymbol{2}}$ **is missing.** | | | | | | | | | |
| 20% missingness + weak association | -0.0298 | 0.0525 | 0.0512 | 0.0036 | 0.898 | -0.0268 | 0.0510 | 0.0512 | 0.0033 | 0.929 |
| 20% missingness + strong association | -0.1021 | 0.0507 | 0.0518 | 0.0130 | 0.501 | -0.0995 | 0.0496 | 0.0517 | 0.0124 | 0.521 |
| 40% missingness + weak association | -0.0456 | 0.0609 | 0.0616 | 0.0058 | 0.892 | -0.0402 | 0.0615 | 0.0613 | 0.0054 | 0.891 |
| 40% missingness + strong association | -0.1460 | 0.0609 | 0.0629 | 0.0250 | 0.338 | -0.1451 | 0.0632 | 0.0627 | 0.0251 | 0.355 |
|  | **m-DAG 3a:** $\boldsymbol{O}_{\boldsymbol{2}}$ **missing dependent on** $\boldsymbol{A}_{\boldsymbol{1}}$ **and** $\boldsymbol{O}_{\boldsymbol{1}}$**;** $\boldsymbol{Y}$ **missing if** $\boldsymbol{O}_{\boldsymbol{2}}$ **missing.** | | | | | | | | | |
| 20% missingness + weak association | -0.0001 | 0.0499 | 0.0502 | 0.0025 | 0.948 | 0.0027 | 0.0491 | 0.0501 | 0.0024 | 0.946 |
| 20% missingness + strong association | -0.0015 | 0.0494 | 0.0499 | 0.0024 | 0.953 | 0.0023 | 0.0499 | 0.0498 | 0.0025 | 0.953 |
| 40% missingness + weak association | -0.0009 | 0.0595 | 0.0566 | 0.0035 | 0.944 | 0.0035 | 0.0571 | 0.0565 | 0.0033 | 0.949 |
| 40% missingness + strong association | 0.0002 | 0.0552 | 0.0580 | 0.0031 | 0.970 | 0.0003 | 0.0563 | 0.0578 | 0.0032 | 0.969 |
|  | **m-DAG 3b:** $\boldsymbol{O}_{\boldsymbol{2}}$ **missing dependent on** $\boldsymbol{A}_{\boldsymbol{1}}$**,** $\boldsymbol{O}_{\boldsymbol{1}}$**and a common cause between** $\boldsymbol{M}_{\boldsymbol{O}\boldsymbol{2}}$**and** $\boldsymbol{Y}$ **which induces an association between** $\boldsymbol{M}_{\boldsymbol{O}\boldsymbol{2}}$**and** $\boldsymbol{Y}$**;** $\boldsymbol{Y}$ **missing if** $\boldsymbol{O}_{\boldsymbol{2}}$ **missing.** | | | | | | | | | |
| 20% missingness + weak association | -0.0011 | 0.0502 | 0.0497 | 0.0025 | 0.944 | 0.0013 | 0.0476 | 0.0497 | 0.0023 | 0.954 |
| 20% missingness + strong association | -0.0005 | 0.0494 | 0.0493 | 0.0024 | 0.949 | 0.0029 | 0.0490 | 0.0493 | 0.0024 | 0.950 |
| 40% missingness + weak association | 0.0013 | 0.0551 | 0.0566 | 0.0030 | 0.950 | 0.0033 | 0.0565 | 0.0564 | 0.0032 | 0.950 |
| 40% missingness + strong association | -0.0011 | 0.0546 | 0.0547 | 0.0030 | 0.954 | 0.0021 | 0.0539 | 0.0547 | 0.0029 | 0.949 |
|  | **m-DAG 4a:** $\boldsymbol{A}_{\boldsymbol{2}}$ **missing dependent on** $\boldsymbol{O}_{\boldsymbol{2}}$ **and** $\boldsymbol{Y}$ **missing if** $\boldsymbol{A}_{\boldsymbol{2}}$**missing.** | | | | | | | | | |
| 20% missingness + weak association | -0.0006 | 0.0500 | 0.0490 | 0.0025 | 0.950 | 0.0022 | 0.0487 | 0.0490 | 0.0024 | 0.949 |
| 20% missingness + strong association | -0.0012 | 0.0502 | 0.0499 | 0.0025 | 0.944 | 0.0021 | 0.0492 | 0.0498 | 0.0024 | 0.956 |
| 40% missingness + weak association | -0.0011 | 0.0591 | 0.0574 | 0.0035 | 0.945 | 0.0027 | 0.0564 | 0.0572 | 0.0032 | 0.955 |
| 40% missingness + strong association | 0.0004 | 0.0564 | 0.0574 | 0.0032 | 0.952 | 0.0014 | 0.0563 | 0.0574 | 0.0032 | 0.954 |
|  | **m-DAG 4b:** $\boldsymbol{A}_{\boldsymbol{2}}$ **missing dependent on** $\boldsymbol{O}_{\boldsymbol{2}}$ **and** $\boldsymbol{Y}$ **(assuming a common cause of missingness in** $\boldsymbol{A}_{\boldsymbol{2}}$ **and** $\boldsymbol{Y}$**);** $\boldsymbol{Y}$ **missing if** $\boldsymbol{A}_{\boldsymbol{2}}$ **missing.** | | | | | | | | | |
| 20% missingness + weak association | -0.0266 | 0.0495 | 0.0499 | 0.0032 | 0.915 | -0.0238 | 0.0491 | 0.0498 | 0.003 | 0.935 |
| 20% missingness + strong association | -0.0815 | 0.0496 | 0.0502 | 0.0091 | 0.632 | -0.0785 | 0.0488 | 0.0500 | 0.0086 | 0.658 |
| 40% missingness + weak association | -0.0370 | 0.0559 | 0.0562 | 0.0045 | 0.893 | -0.0352 | 0.0575 | 0.0560 | 0.0045 | 0.909 |
| 40% missingness + strong association | -0.1124 | 0.0589 | 0.0593 | 0.0161 | 0.548 | -0.1066 | 0.0596 | 0.0591 | 0.0149 | 0.570 |

Footnotes: ^a^ Treatment effect 5 simulation setting when there is no treatment effect at stage 2 for half of the participants, but a relatively large effect for the other half.

^b^ $O_{1}$= basic characteristic; $A_{1}$= stage 1 treatment; $O_{2}$= stage 1 responder status; $A_{2}$= stage 2 treatment; $Y$ = stage 2 outcome

^c^ For weak association between the missing indicator and other variables (as described below) an OR of 1.6 was used; and for a strong association an OR of 3 was used. The other variables were: missing data scenario 2a) $A_{2}$→$M_{Y}$ ; 2b) $Y$ →$O_{2}$ and $A_{2}$→$M_{Y}$; 3a) $A_{1}$→$M_{O2}$ and $O_{1}$→$M_{O2}$; 3b) $A_{1}$→$M_{O2}$ , $O_{1}$→$M_{O2}$ and $Y$ →$M_{O2}$; 4a) $O_{2}$→$M_{A2}$; and 4b) $O_{2}$→$M_{A2}$ and $Y$ →$M_{A2}$.
